# Can an old rook learn new tricks? Vocal command comprehension and obedience in rooks (*Corvus frugilegus*)

**DOI:** 10.1007/s10071-025-02002-8

**Published:** 2025-09-09

**Authors:** Francesca M. Cornero, Willa M. Lane, Nicola S. Clayton

**Affiliations:** Department of Psychology, University of Cambridge, Downing Street, Cambridge, CB2 3EB UK

**Keywords:** Birds, Rooks, Command obedience, Comparative cognition, Intraspecific communication, Corvid communication

## Abstract

Although many animal species are known to learn to respond to human verbal commands, this ability is understudied, as are the cues used to do so. For the best-studied species, the dog, domestication itself is used to justify successful attending to human communicative cues. However, the role of domestication in sensitivity to human cues remains debated. Corvids are songbirds that engage in complex communicative behaviors with conspecifics and with other species. Rooks (*Corvus frugilegus*) are a cognitively complex, undomesticated corvid shown to possess complex vocal behaviors, and to learn to follow human gaze and pointing cues. Leonidas (Leo), an adult rook, began following verbal commands during unrelated studies, and was tested to confirm his command proficiency and to examine what part(s) of the signal he was using. Leo learned to perform three verbal commands correctly, and was primarily attending to the auditory components of the commands. He showed no evidence of using gaze or any subconscious cues from the experimenter, and though he did initially attend to lip movements, he rapidly overcame their absence. Two additional rooks could only be tested for a shorter duration, but also showed learning of commands, and reached proficiency in some. The ability of this undomesticated non-mammalian species to learn human verbal commands joins existing evidence that neither domestication nor extensive enculturation are strictly necessary for use of heterospecific human cues: instead, some species may be able to co-opt other complex cognitive abilities, possibly the same ones required for their complex communication, to achieve this.

**Supplementary Information:**

The online version contains supplementary material available at 10.1007/s10071-025-02002-8.

## Introduction

In comparative cognition, a command can be defined as a human-given signal intended to produce a predictable response in a target individual (i.e. Mills 2005): commands may be auditory (either verbal or through other sounds), gestural (including pointing or other motions), or even tactile (as is the case, for example, for horses under saddle). A wide range of non-human animals can learn human commands. Pet dogs are the ubiquitous example, though many non-domesticated species can also functionally respond to human commands. These include zoo animals used in educational “shows”, rescued wildlife used in outreach programs, and captive animals trained to obey caretakers to facilitate management or administer medical care (e.g. Brando & Norman 2023; Spooner et al. 2021; Ward & Melfi 2013; Young & Cipreste 2004). Compared to soundless gestures, when a human gives a vocal command, much more information is available to an animal than just the words spoken: the entire command signal includes body posture, conscious or subconscious physical cues or gestures, eye-gaze cues, intonation and pitch changes, speaker-specific information such as biological sex and accents, and more (Braem & Mills 2010; Fukuzawa et al. 2005a; Mills 2005). For example, in the widely-studied domestic dog, dogs’ responses to vocal commands were affected by tone, auditory quality, individual phonemes, accompanying physical cues (such as gaze, body posture, gestures, distance from the animal, etc.), and context of when and how commands were given (e.g. Fukuzawa et al. 2005a, b; Mills et al. 2005). Therefore, the extent to which the isolated *auditory* component of a command signal is used for choosing a response behavior by animals, especially when any human speech is involved, remains unclear.

For instance, in a study investigating the effects of commonly co-occurring cues on response accuracy, dogs’ performances decreased when a voice recorder was used, in certain contexts in which sunglasses were used (eliminating eye cues), and when the experimenter’s distance increased and visibility decreased, but not when the experimenter gave commands from a sitting rather than a standing position (Fukuzawa et al. 2005a). Likewise, in another study, dogs were less accurate at responding to commands when the trainer was missing and commands were heard through a speaker, but not when a visual projection of the trainer was seen giving the commands (Pongrácz et al. 2003). Additionally, the emotional tone used to give commands affected the reliability of responses, with gloomy or angry tones leading to performance decreases in some cases, and “informative” tones were better obeyed than “imperative” ones (Mills et al. 2005; Scheider et al. 2011). The natural correlation between the pitch and timbre of the human voice was also important: performance suffered when pitch and timbre were manipulated to be unrealistic (as corresponding to either the male or female sex), but not when they were consistent with reality (even when artificially-generated to sound male from an originally female trainer voice; Sturdy et al. 2022). A further study assessing the effect of changes on phoneme-like components of known vocal commands found that all phoneme changes affected dogs’ responses, suggesting that dogs perceive a vocal command cue holistically rather than paying attention to only specific parts of a word (Fukuzawa et al. 2005b).

When domestic dogs succeed at human communicative tasks, including gaze-following and pointing gestures in additional to vocal commands, both domestication itself and dogs’ extensive exposure to humans during their lives are employed as explanatory variables. Pet dogs are thought to have become more selectively sensitive to human signals and communication methods through the domestication process than their ancestors or other animals, but they also have significant experience with human communicative cues when raised as pets in a home (Fukuzawa et al. 2005a; Gibson et al. 2014; Mills et al. 2005; Soproni et al. 2001; Sturdy et al. 2022). It is often argued that dogs outperform most, if not all, other animals at tasks requiring understanding of human communicative signals and cues, such as pointing or gaze-following (Hare et al. 2002; Scheider et al. 2011; Soproni et al. 2001; Udell et al. 2010). It is intensely debated whether this is evidence of a unique sensitivity that was either intentionally bred-for, or is a side-effect of, the domestication process of dogs from a wolf-like recent ancestor (Andics et al. 2016; Boros et al. 2024; Hare & Tomasello 2005; Miklósi et al. 2004; Soproni et al. 2011; Virányi et al. 2004; Range & Virányi 2015; Gibson et al. 2014; see Marshall-Pescini et al. 2017; Range & Marshall-Pescini 2022; Udell et al. 2010 for discussions).

Unfortunately, there are few published instances of empirical investigation into the factors affecting obedience to human verbal commands in non-domestic mammal species. One captive Steller sea lion that had learned 10 commands was able to respond correctly both when different trainers gave the commands (regardless of biological sex) and when commands were played from a recording (although in the recorded version, some variation existed in responses to different trainers; Sasaki et al. 2022). Additionally, when command context was manipulated for a captive walrus with prior experience on 10 vocal commands, no differences between different experimenters were noted. The walrus responded correctly (albeit with some variation) when commands were given directly, when the trainer was covered with a cloak and goggles to remove physical cues, when the trainer was out of sight, and when the trainer was out of sight and a recording was used (Endo et al. 2020). Conversely, organutans (and possibly other apes) may prefer gestural information, as evidenced by a zoo study assessing comprehension of previously-trained cues in orangutans; the orangutans were highly accurate with both the entire instructional signal (including gestures, gaze, and vocal instructions) and with gestures alone, but their performance suffered with either eye gaze or vocal instruction alone (Dezecache et al. 2019).

However, it is clear, from these case studies and from the success of animal shows featuring a variety of mammalian species (frequently, dolphins, whales, and pinnipeds), that several species can learn to respond to human commands accurately. Compellingly, dolphins have shown complex comprehension of human instructions, including those requiring detailed actions that have to be carried out in specific orders or manners to be correct (although these efforts used learned artificial sounds rather than human speech; Herman et al. 1984). More broadly, some effort has also been expended on investigating how mammals process human sounds aside from learned speech commands: house cats, for instance, learned to discriminate their own name from other nouns and from other cats’ names, but café cats did not, suggesting experience and learning are required for this discrimination (Saito et al. 2019). On the other hand, tonal differences, when paired with unlearned words, are unlikely to be instinctually meaningful to domestic horses, as they showed no behavioural or physiological differences in an aversive task when either soothing, praising voices or harsh, dissuasive tones were used (Heleski et al. 2015). This suggests there may be species-specific variation in the propensity to comprehend or learn human auditory communicative signals, but the amount of controlled studies conducted remains low, and procedural variations may affect the comparison of results. Existing results that do include non-domestic species responding to human commands or communicative signals suggest that domestication may not be necessary for this ability. Could other underlying cognitive abilities be at play in the co-opting of heterospecific vocal cues such as human commands in some nonhuman species?

Even less is known about the ways in which non-mammalian animals may process, learn, and use human language signals, even though some birds, such as parrots and many corvids, are capable of producing human speech sounds through mimicry. The ability of African grey parrots to respond to, reproduce, and use human speech labels successfully and accurately is well-documented and studied (using the model/rival technique, see Pepperberg 1985; 1992; 1994; 1999; 2002; 2013; Pepperberg et al. 1999). However, little to nothing is known about what corvid and other parrot species may be picking up from exposure to human speech sounds, even though there is evidence that a number of these species can also engage in vocal mimicry (e.g. Wascher et al. 2025). Separately, birds have been taught commands in order to assess some other capacity experimentally in a handful of unrelated instances. Crows were taught to vocalise according to cues indicating to either vocalise or remain silent, and did so successfully, displaying evidence of flexible control over vocalisations (with “commanded” vocalisations shorter and less variable than spontaneous ones, Brecht et al. 2019). Crows have also been successfully taught to vocalise a certain number of times in accordance with both visual and auditory cues (Liao et al. 2024). Finally, blue-throated macaws were taught to perform a few behaviours on command, with the goal of then teaching a “repeat” command to assess their memory for their own actions: not only did they learn this command, but they were able to repeat their own behaviours successfully after short intervals, on occasion even generalising to novel behaviours (Torres Ortiz et al. 2022). There is therefore some experimental evidence, in addition to that from avian animal “shows” (which often include parrot, corvid, and raptor species; Spooner et al. 2021), that at least some bird species can learn to use human commands. However, it remains unknown how birds learn to use human commands and what cues they primarily pay attention to when doing so.

Corvid species may be particularly well-suited for investigations into the interspecific comprehension of human vocal cues. Species from the corvid family are songbirds known to be cognitively complex and to possess abilities often rivalling those of great apes, including mental-time travel, means-end reasoning, tool-use, mental-state attribution, and more (e.g. Baciadonna et al. 2021; Emery & Clayton 2004; Lambert et al. 2019, among many others). However, in addition to their other cognitive abilities, corvid species also display complex communication systems, including the co-opting or functional use of communications from heterospecific species. Corvids are songbirds with complete “song systems” and, like other songbirds, they are vocal learners, meaning their vocalizations must be learned from their peers (Wascher & Reynolds 2025); additionally, many species have been shown to be open-ended vocal learners, able to learn vocalizations into adulthood, including mimicry of other species and of humans (Liao et al. 2025; Wascher et al. 2025). They use their vocalizations for several important communicatory functions: for instance, ravens can recruit other ravens to previously-discovered food that is being monopolized by individuals (Heinrich 2014), vocalize in specific ways to reduce or minimize conflict (Bugnyar 2024; Szipl et al. 2017), and even exploit conspecific signals, such as those from wolves or human gunshots, to obtain food (Vucetich et al. 2004; White 2005). Work on several corvid species has demonstrated that there is predictable individual variation in contact calls or other vocalizations, which may serve as personally-identifiable information or as a manner of conspecific recognition (e.g., Boeckle & Bugnyar 2012; Kondo et al. 2010a, b, 2012; Martin et al. 2022, 2024; Sierro et al. 2020; see Wascher & Reynolds 2025). Some corvids have also demonstrated the ability to convey referential-like information in their communication. This includes ravens communicating information regarding food type (Bugnyar et al. 2001; Marzluff et al. 1996), American crows communicating information about individual threatening humans (Cornell et al. 2012; Marzluff et al. 2010), Siberian jays conveying specific behaviours of hawk predators in predator alarm calls (Griesser 2008, 2009), and many other corvid species communicating differential information about types of predators and/or the risk level posed by predators (Billings et al. 2017; Dahl & Ritchison 2018; Griesser & Ekman 2005; Iglesias et al. 2014; Stone & Trost 1991; Yorzinski & Vehrencamp 2009).

In corvids, the use of vocal communication may be under cognitive control, and may be used by, or contribute to, other complex cognitive functions (Liao et al. 2025). For instance, ravens both infer dominance information from conspecific vocalizations and display violation of expectations to such dominance predictions (Massen et al. 2014), Siberian jays “trust” the alarm calls of known conspecifics or cooperative partners over unknown conspecifics that may be deceitful (Cunha & Griesser 2021), and carrion crows learn to vocalize or remain silent in response to visual stimuli including by vocalizing a specific number of times (Brecht et al. 2019; Liao et al. 2024). In songbirds including corvids, complex communicative abilities and vocal learning have been associated with improved problem-solving abilities (Audet et al. 2023), and may be interconnected with specialized neural pathways required for other advanced cognitive abilities (see Liao et al. 2025; Wascher & Reynolds 2025). Additionally, the social complexity hypothesis connects social complexity with communicative complexity, as flexible and dynamic communication may be required for the maintenance of complex social relations, which in turn may also necessitate general cognitive complexity (see Martin et al. 2024). Given that several species of corvids demonstrate complex communicative abilities in addition to complex social and cognitive abilities, including some evidence of exploiting heterospecific communication, this group of birds may be uniquely well-suited for assessing the possibility that underlying communicative and/or cognitive abilities may be able to be co-opted for successful interpecific communication with humans.

During unrelated experimental habituation and training (see Cornero & Clayton 2025), one adult rook (*Corvus frugilegus*), Leo, began to show evidence of attending to some command signals given by the main experimenter (FMC). Although rooks have been presented with relatively fewer cognitive tasks than other corvid species (e.g. Baciadonna et al. 2021; Clayton & Emery 2005; 2007; 2015), they have demonstrated remarkable competencies, including tool-use, tool-modification, metatool-use, cooperative problem-solving with string-pulling, physical cognition, and complex social abilities such as individual recognition, social foraging and food-sharing, and post-conflict affiliation (Bird & Emery 2008; 2009a,b, 2010; Dally et al. 2008; Emery et al. 2007; Jolles et al. 2013; Seed et al. 2006; 2007; 2008; Tebbich et al. 2007). As members of the corvid family, they are open-ended vocal learners (Liao et al. 2025); while a recent model-based approach did not find evidence of vocal mimicry in rooks (Wascher et al. 2025), anecdotal evidence for limited vocal mimicry in some rook individuals does exist, including in laboratory settings (Martin et al. 2024; Woolfson 2008; Martin, K., personal communications 2022 and 2025). There is some evidence they may attempt to “sing-along” to heterospecific sounds, including music, by modifying their song according to auditory stimuli (Martin et al. 2025a), and their calls have identifiable individual variation (Martin et al. 2022), including over an individual’s entire repertoire (Martin et al. 2023). Although little else is known about rook conspecific vocal communication to date, their high social complexity and the vocal behaviors that have been studied, as well as their advanced cognition, suggest they may be ideal candidates for the study of interspecific communication with humans. Rooks have also demonstrated the ability to learn to use some forms of human gestural and gaze cues for locating food or solving string-pulling tasks (Schmidt et al. 2011), and to co-orient their gaze with humans in some situations (Schloegl et al. 2008). Therefore, when Leo’s behaviour was noted and recognised, it was thought it might indeed reflect functional use of the experimenters’ communicative signals to obtain food rewards, rather than accidental actions taken by the rook and corresponding with the instructions by chance, or subconscious direction or cueing by the experimenter.

Rooks are a wild, undomesticated species of songbird, and as such, their successful use of human communicative cues would further suggest that domestication is, at least, not *necessary* for a nonhuman to functionally make use of human communicative cues. Additionally, although Leo was hand-raised and subsequently lived in lab settings, his experience cannot be considered at all comparable to that of an “enculturated” nonhuman, such as those raised in a human home (pet dogs) or extensively socialised in lab settings for experimental purposes. Although human experimenters and animal care technicians were familiar to Leo from other experiments and husbandry needs, he lived in a large, outdoor aviary in a conspecific social group, and spent the vast majority of his life among other rooks, with human interactions being of a largely observational nature (rather than directly interactive at close range). Although it already appears likely that intentional domestication is not *required* for functional following of human-given cues, dedicated attempts to unearth such abilities in non-domesticated animals have rarely been conducted in an experimental, well-controlled manner with the use of human vocal commands; additionally, little to no such efforts have been documented, scientifically, involving obedience to human vocal cues by non-mammalian species (but see Pepperberg 1990; 2012a,b; 2014, among others, for a large body of work documenting referential vocal communication with African grey parrots). Can another avian species known to be cognitively complex, for which one individual spontaneously appeared to show aptitude in learning commands, be studied in such a stringent manner, and provide robust documented evidence of success? If so, can the components of the auditory human command signal being used by the bird be studied and isolated? This study attempts to answer both questions. Functional competence at using human communicative cues in Leo and other rooks would suggest, as others have posited (e.g. Kriengwatana et al. 2015), that domain-general cognitive abilities, and/or existing underlying communicative abilities used for both conspecific or heterospecific communication in the wild, can be co-opted to functionally utilise human communicative signals.

## Methods

### Subjects

The total sample was seven adult rooks (16–21 years old at the start of testing): Leo, Connelly, Aristotle (males); Fry, Bussell, Cassandra, and Huxley (females). However, Cassandra, Aristotle, Bussell and Huxley were unwilling to work for this experiment: Bussell and Cassandra never remained in the compartments with the experimenter long enough to be tested on commands, Huxley was no longer able to remain in testing compartments after the death of her mate Plato (see Cornero & Clayton, 2025), and Aristotle was not willing to land on the table (which was required to perform certain commands) during the course of the study. Rooks lived as a group in a large outdoor aviary (20 × 10 × 3 m), with smaller indoor compartments (3 × 1 × 2 m) connected by hatches (0.5 × 0.5 m) being used for testing. For Leo, testing began in September 2021 and lasted until the end of January 2024; for Fry and Connelly, testing began in June 2022 and February 2023, respectively, after they learned the basic command behaviours. Fry and Connelly were tested until March 2024 due to changes in main experimenter availability. Please note that information regarding Fry and Connelly’s experimental experiences and results are to be found in the *Supplementary Materials* due to methodological differences and incomplete testing due to time constraints. Rooks were taken from wild colonies around the laboratory as nestlings between the years 2002–2006 with appropriate Natural England licenses, were hand-raised by NSC and members of her lab, and had subsequently lived in the laboratory. Several individuals had participated in some of the experiments mentioned above (e.g. Jolles et al. 2013; Seed et al. 2006; 2007; 2008; Tebbich et al. 2007), particularly Connelly and Fry. This experiment was approved by the University of Cambridge (AWERB Sub-Standing Committee) as a non-regulated procedure (NR2023/45) and followed Home Office Regulations and the Association for the Study of Animal Behaviour’s Guidelines for the Treatment of Animals in Behavioural Research and Teaching.

A long habituation period (to experimenter FMC, testing compartments, and general experimental procedures) was necessary before birds became willing to approach and work (see Cornero & Clayton 2025). Subsequently, individuals were visually isolated from other rooks when possible during testing sessions, which lasted for as long as a subject was willing to participate and always began and ended by the bird voluntarily approaching or leaving the testing area. Although the rooks being tested could not be shut inside the testing compartments for visual isolation, rooks in the aviary could not observe trials unless they approached the area immediately outside the compartment, and if so, the experiment was paused. Birds were never food or water deprived: they were fed a maintenance diet of soaked cat biscuits, vegetables, seeds, fruit and hard-boiled eggs. Waxworm larvae, a highly preferred food not otherwise available, were used as rewards.

### Apparatus

**Fig. 1:**
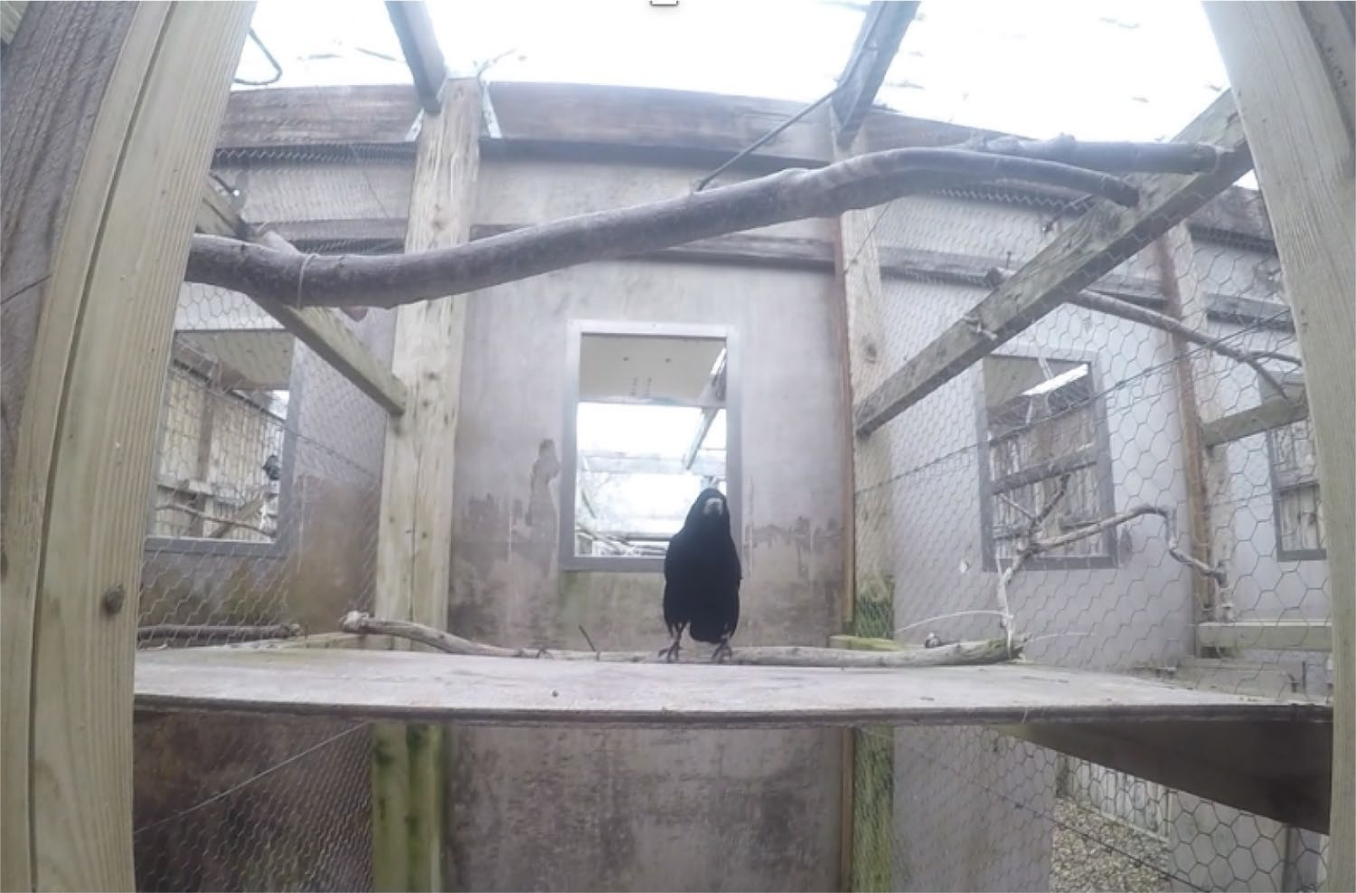
The testing compartment used for command trials, including Leo (pictured) standing at his starting position, the perch behind the testing surface. Note also the higher perch, suspended above the table, which was a possible “Up” response location

Rooks were tested on a wooden board, ~ 115 × 60 cm, placed at experimenter’s chest height (when standing on a footstool, Fig. 1). All trials were recorded with a GoPro Hero4 video camera strapped to FMC’s chest. When command recordings were utilised, they were both recorded on and were subsequently played from an iPhone X. iMovie (MacIntosh) was used for audio track manipulations (increasing volume), and when required, conversion to video format (for command titles to be visible to the experimenter). Statistical analyses and data visualization were carried out with RStudio (RStudio Team 2020).

### General procedure

Over the course of habituation to the main experimenter (FMC) and testing for an unrelated object permanence experiment (see Cornero & Clayton 2025), one rook, Leo, appeared to learn to respond to a handful of experimenter-given commands, more or less accidentally. He spontaneously acquired “Come here” as a cue to approach the experimenter because it led to rewards. He was instructed to “Wait!” by the experimenter during some tasks in which he was attempting to choose before manipulations were complete (and was subsequently intentionally reinforced on “Wait!” during preparation for an unrelated reasoning by exclusion experiment, in prep.). He was taught “Speak” during breeding season as part of an effort to maintain habituation and cooperation during this period, as he refused to work on other tasks. When the main experimenter noted that Leo appeared to be responding to each command correctly, rather than producing a behaviour at random, experimental testing was designed to confirm his performance and to investigate what cue, or cues, he may have been using to respond.

During this initial, pre-trial acquisition period, commands were not presented in the same visual manner, as no testing had been planned. “Come here” was called out from a distant aviary location with the presentation of a worm, “Wait!” was used to keep Leo on the testing branch while manipulations occurred (usually, with one experimenter’s hand held upright), and “Speak” was given from a short distance away, with the experimenter facing the rook and presenting a worm. Before formal assessments could be conducted, it was necessary to ensure Leo was willing to respond to commands presented in the same visual manner (described below, see *Basic Command Presentation*), to determine if he could respond to auditory-only commands, in absence of corroborating visual cues. This required training in the testing compartment to transition Leo’s prior informal experiences to formally waiting on the testing perch and listening for an auditory (only) instruction. Due to unrelated time constraints (a rapidly-impending, but later avoided, closure of the testing site and rehoming of the research animals; see George 2022), it was decided to induce this transition as rapidly as possible. This was therefore done through non-random repetitions of the given commands, behavioural shaping, and assessment of Leo’s current proficiency level in choosing the next commands to be given. As such, the main experimenter (FMC) assessed live what errors Leo appeared to be making, what repetitions or procedures seemed to induce more correct responses, and what commands to present next. It has been shown that training techniques utilising components of human social learning theory engender more effective and rapid learning than trial-and-error learning in cognitively-complex birds, such as African grey parrots (see Pepperberg 1985; 1991; 1992; 1994; 1997; 2013); as such, active training according to these considerations was used here. However, this does not allow for an assessment of Leo’s un-guided learning rates for the commands, and thus only the effects of subsequent alterations to the command signal can be studied and reported. Because Leo had already experienced previous unrecorded learning of each command, he was likely not learning these commands de novo, but simply transferring them to a testing situation (as in many studies in which dogs were previously familiar with commands, i.e. Fukuzawa et al. 2005a, b; Mills et al. 2005; Pongrácz et al. 2003; Sturdy et al. 2022). Once Leo was willing to perform all behaviours at least some of the time with the same visual presentation (see *Basic Command Presentation*), proficiency testing began in the form of the Baseline condition.

Additionally, during Leo’s Baseline condition, a fourth command was introduced: “Up”. This was because Leo had achieved proficiency at “Come here” and “Speak” by that point, but did not appear to be improving at “Wait!”. “Up” was introduced as an alternative third command due to the time pressure of the testing site being scheduled to close and the logically-higher difficulty of “Wait!” (it requires, in addition to choosing the correct response, maintaining delayed gratification for several seconds to avoid retrieving the worm and inducing an error). After a short period of repeated shaping, it was incorporated into the Baseline set and randomised between the other commands, with the intent of dropping “Wait!” and proceeding with “Up” if he achieved proficiency with it. However, Leo then rapidly achieved proficiency on “Wait!” before learning “Up”. It was decided to move on to further testing at that point. “Up” continued to be intermixed between the other commands but proficiency at “Up” was not required for progression to further stages. Because Leo never became proficient at “Up” over the course of the study, “Up” is excluded from statistical analyses.

#### Basic command presentation

All commands were presented by the experimenter in the same visual way. At the beginning of each trial (one command), the rook was asked to land on the perch behind the testing table by being shown the worm. When the rook landed there and looked at the worm, the experimenter set the worm down centrally on the testing surface. For Leo, this was at arm’s length, so that FMC could prevent frequently-attempted worm theft. As soon as the worm was placed on the testing surface, the experimenter called out the pre-determined command while removing her hand from the worm. If the rook performed the correct behaviour, it was allowed to eat the worm. For “Come here” and “Wait!” (after “Go!”), the rook was allowed to jump on the surface and retrieve the worm. For “Speak”, the experimenter picked up the worm and threw it at the rook. For “Up”, the experimenter picked up the worm and handed it to Leo. If the rook performed an incorrect behaviour, the experimenter said “no!” and removed the worm. When a rook erred, the rook’s response was marked as “incorrect”, but further correction trials of the same command were given until the rook performed a correct response. This was done to prevent rooks from choosing to avoid specific commands, i.e., erring “on purpose” to move on to “less aversive” options: for instance, Leo could conceivably choose to never wait if there was no “penalty” for erring and easier commands (and faster worms) could be received by aborting “Wait!” trials. Corrections were repeated until the rook succeeded: when it did, it was allowed to consume the reward but the response recorded remained incorrect. If mistrials occurred (see *Mistrial Procedure* below), trials were repeated directly. Vocal commands were intended to be naturalistic, and as such included differing pitch, intonation, word duration, and other prosodic characteristics of normal human speech. During initial trials, the experimenter attempted to use the same pitch, tonal changes, and word duration that had been chosen for each command during each delivery, but some of these variables were intentionally manipulated in later experiments (in prep.).

For each condition, command testing was divided into “rounds” with 15 trials of each command, for a total of 60 trials per round for Leo. The order of the commands was pseudo-randomised with Random.org such that no more than two of the same command were presented in a row. In order to move on to further conditions, rooks had to obtain a “rolling average” passing criterion of 12/15 correct, on average, for *all* commands simultaneously over three successive rounds of trials, which is significant at the binomial level, with chance at 0.5, for each command (12/15, p = 0.035, two-tailed).^1^ When rooks did not achieve this criterion, even at just one command, further rounds of the same condition were offered until they passed. As such, the minimum number of rounds in which any condition could be passed was 3, even if rooks achieved proficiency earlier. This average-based criterion was chosen to account for normal behavioural and motivational variations that could occur from animal subjects on any given day; these criteria were also more stringent than corresponding dog studies (e.g. compared to Fukuzawa et al. 2005a, b; Sturdy et al. 2022). When criterion was attained on all commands, the number of rounds taken to pass the condition was noted, and the next condition was offered. Rooks could participate for as long as they wanted. Rooks were tested 5 days a week and could choose to return for testing multiple times in the same day.

#### Command descriptions

We now describe each command’s required response and correct/incorrect determination criteria (see also online *Supplementary Videos*). Critically, behavioural responses were intended to be scored so as to be mutually exclusive: unless a mistrial occurred, the rook’s behaviour after a command could always be categorised as one of the possible responses.

**Fig. 2:**
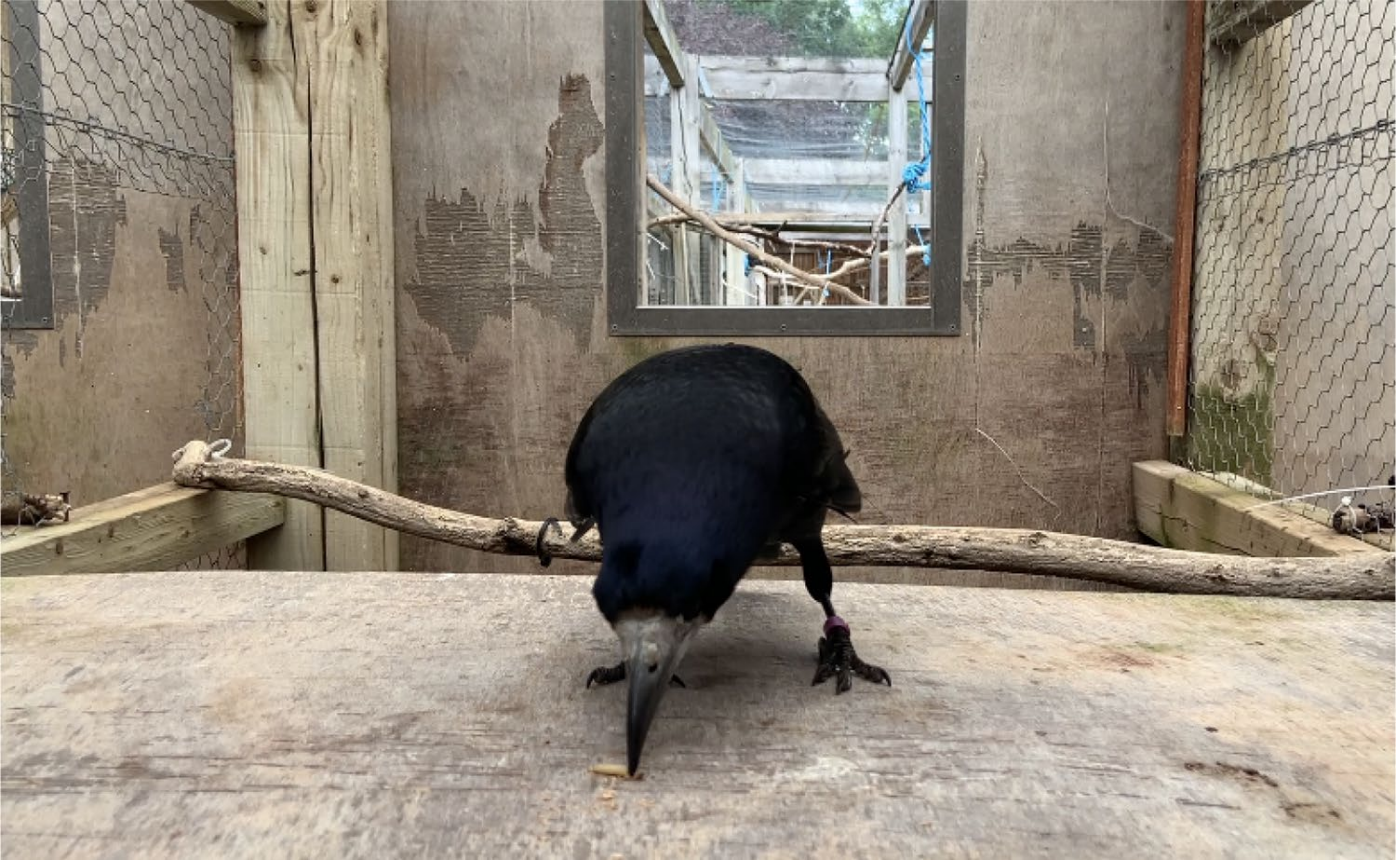
Leo demonstrating the “Come here” behaviour, from the experimenter’s perspective: when correct, he would land onto the table and retrieve the worm himself within the allotted time frame

*“Come here”*: In order to succeed, the rook had to begin approaching to retrieve the worm within 3 s of hearing the command (as counted in the experimenters’ head, to minimise physical movements and cues that could accidentally be given if using tools such as stopwatches). If rooks did not begin approaching by this time and instead remained stationary or moved away from the worm, the response was considered to be “Wait!” as no visible attempt had been made to approach. If rooks vocalised, the response was considered to be “Speak”. If the rooks flew to a higher branch, the response was considered to be “Up”. All responses other than approaching the worm within the time-frame were considered to be incorrect. Because Leo made choices rapidly and boldly, he had to jump onto the table within the 3 s time frame to be considered correct – this was the initially determined criterion (see Fig. 2).

**Fig. 3:**
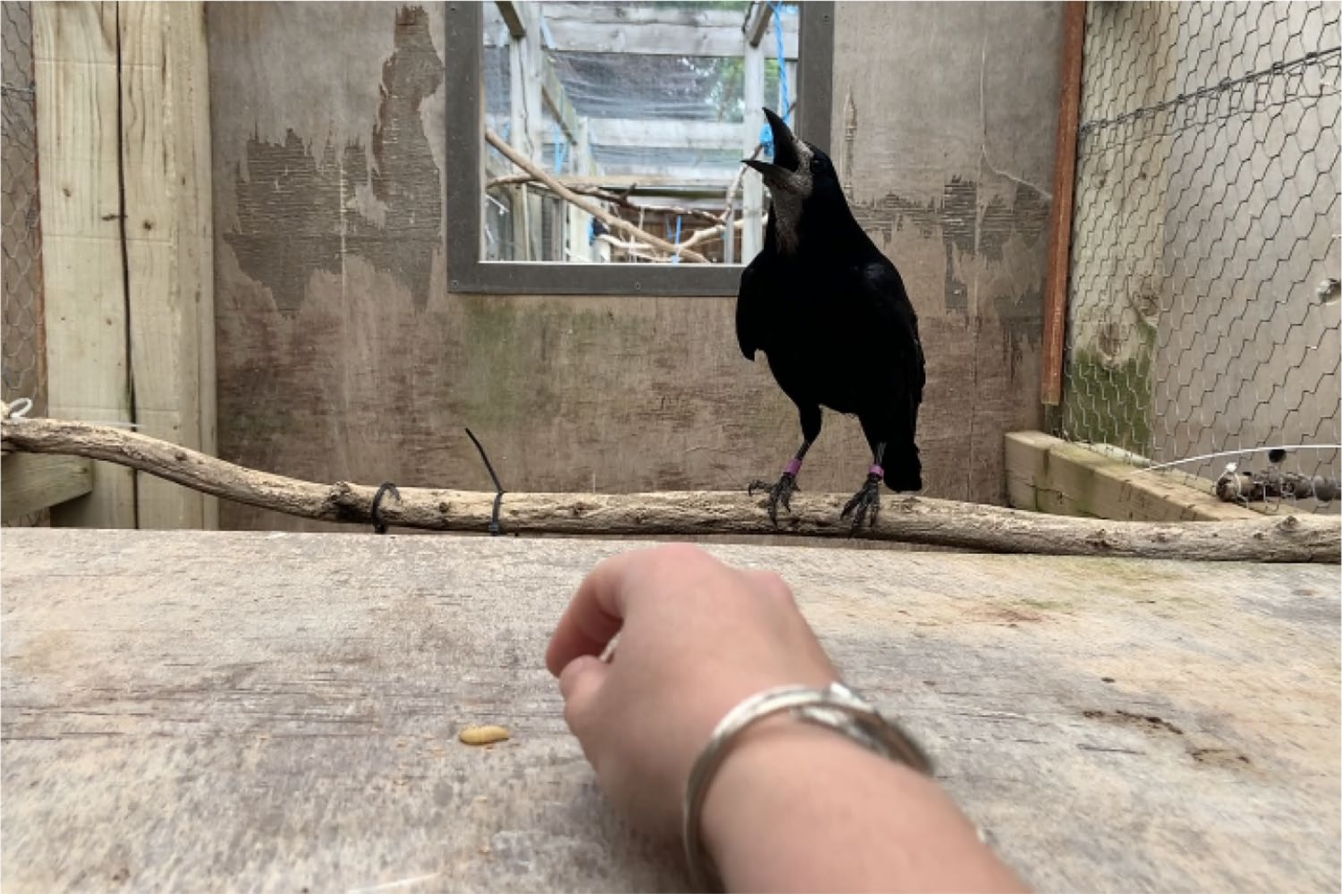
Leo demonstrating the “Speak” behaviour, from the experimenter’s perspective: any type of vocal call would be accepted, but here he performed a full call sequence while the experimenter reached for the worm to give it to him

*“Speak*”: In order to succeed, the rook had to begin to make any vocal noise within 3 s of hearing the command (as counted in the experimenter’s head), in this case without moving towards the worm (see Fig. 3). A singular “caw” or other vocal utterance sufficed, but occasionally entire call sequences were uttered, which were also accepted. Moving towards the worm, failing to begin vocalising in the time frame, and moving to a higher branch were considered to be errors (“Come here”, “Wait!”, and “Up”, respectively). Performing “Speak” in conjunction with any other behaviour, other than remaining motionless, was likewise considered to be an error.

**Fig. 4:**
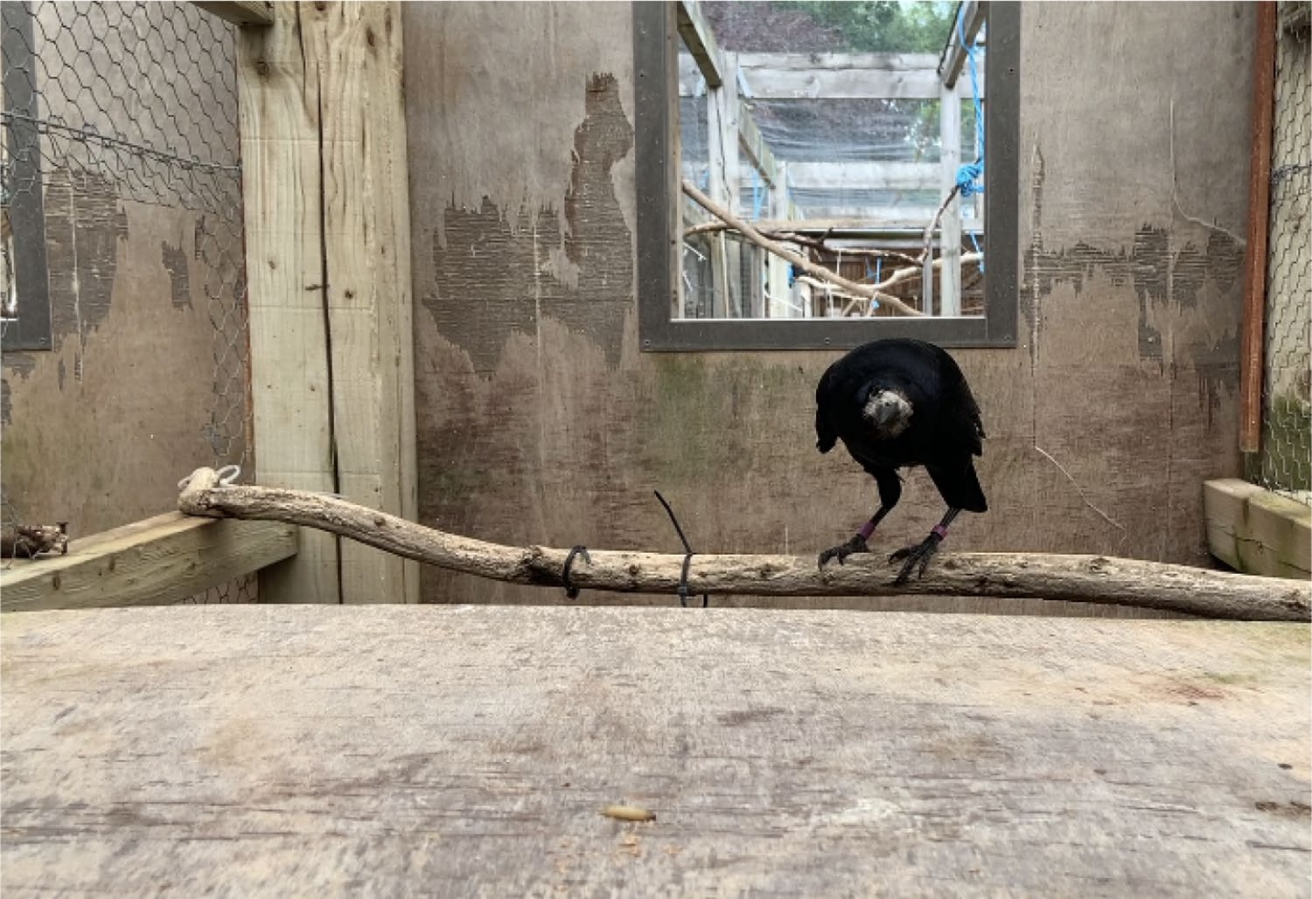
Leo demonstrating the “Wait!” behaviour, from the experimenter’s perspective: when correct, he usually waited poised to jump onto the table to collect the worm (pictured), but did not leave the branch behind the table until he heard “Go!”

*“Wait”*: In order to succeed, the rook had to remain equidistant or move further away from the worm for 5 s after hearing the command (as counted in the experimenter’s head; a longer delay was chosen to make differentiating between responses to “Wait!” and “Come here” unequivocal), only moving towards it to retrieve it when the experimenter said “Go!” at the end of the delay (see Fig. 4). Moving away from the worm without leaving the testing compartment was accepted (if part of a correct response) as it could be a self-distracting behaviour, common during delayed gratification tasks in humans and other animals (see Koepke et al. 2015). Vocalising, approaching the worm in any way, or jumping up to a higher perch (both of which were closer to the worm than the start position) were errors (“Speak”, “Come here”, and “Up”, respectively). The rook did not have to obey “Go!” in order to be correct, as this would therefore require 2 correct responses for one trial; they only had to avoid approaching before hearing “Go!”. However, in the majority of cases, rooks began moving to the reward as soon as they heard the release cue.

**Fig. 5:**
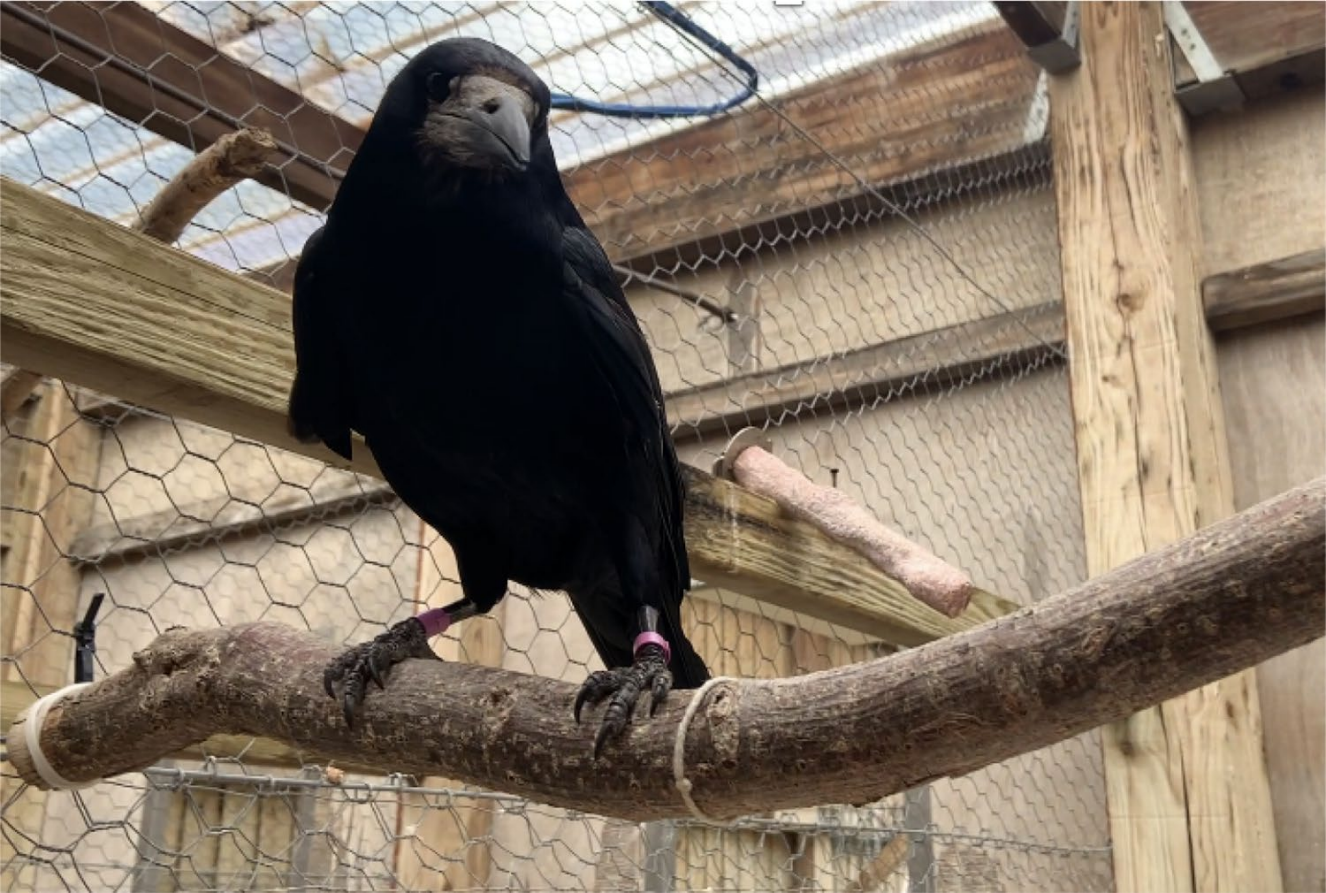
Leo demonstrating the “Up” behaviour, from the experimenter’s perspective: he could choose the pictured perch, suspended above the table, or the pink perch pictured behind it, as possible landing locations to be correct

*“Up”:* In order to succeed, Leo had to fly or jump up to one of two possible branches above the testing surface within 3 s of hearing the command (as counted in the experimenters’ head, see Fig. 5). Vocalising, attempting to take the worm, or failing to move within the delay period were errors (“Speak”, “Come here”, and “Wait!”, respectively). He was, however, allowed to use the table to jump up to the higher perch above it, as long as he bypassed the worm on the table. Note that “Up” proficiency was not required to pass on to future conditions, but the command was offered.

Responses were scored and recorded live by the main experimenter (FMC). Additionally, an interobserver reliability measure was run (see *Mistrial Procedure*, below), and the second experimenter (WML) was present for all Controls (see *Experimental Conditions* below) and for about a third of all experimental sessions from October 2023-March 2024. The second experimenter either always agreed with the main experimenter’s decisions or, if she did not, said so to the experimenter at the time. Both experimenters would then discuss which response had been given by the bird and/or if a mistrial had occurred, and came to joint agreement before recording the responses.

#### Mistrial procedure

If rooks left the compartment abruptly, the trial was considered “not attended” and was repeated; this was judged a failure of motivation or a fear reaction, rather than a failure. Rooks were often scared by sounds, called away by mates, or left to engage in other behaviours (e.g. eat, drink, bathe, food share). Trials were not begun, or marked as mistrials if accidentally begun, if the rook did not appear to be paying attention. Behaviours that indicated this included: 1. Not looking at the experimenter and worm (such as looking around the compartment or into the aviary); 2. Being frightened; 3. Calling to experimenter or other rooks; 4. Preening, napping, regurgitating or eating food, etc. Additionally, mistrials could be declared due to experimenter error, due to unclear behavioural response (for instance, if multiple command response behaviours were performed at once), if rooks attempted to steal the worm before the full command was uttered, when more than two of the same command were accidentally played in a row during Partially-Blinded trials, or when a rook abruptly left the testing compartment after error(s) without remaining long enough to pass correction trials. Trials scored as mistrials were repeated directly. A blinded interobserver reliability measure was run to ensure validity with regards to both the behavioural scoring of the responses and the assessment of mistrials. A student familiar with this study and with the mistrial criteria was given a set of 40 randomly-selected videos, 20 of which had been classified as mistrials (consisting of 38.46% of mistrials at that point in time) and 20 which had not. She was asked to decide, blindly, if a mistrial had occurred and, additionally, what the response behaviour was. Inter-observer reliability was very high, with 18/20 videos of each category correctly assigned (Cohen’s kappa: 0.895). Additionally, the blinded observer classified all 4 trials that had been scored as correct also as correct, as well as an additional 2 trials which had been scored as incorrect by the experimenter, suggesting that the actual scoring used was conservative in assigning correct responses.

#### Experimental conditions

1. *Pilot*: Before testing began, when it appeared that Leo had switched to performing commands in the testing compartment, he received a brief, 3-round Pilot so the main experimenter could assess the functionality of the methodology and, particularly, of distinguishing his behavioural responses. Based on this pilot, the delay for “Wait!” was increased from 3 to 5 s for all further testing, to ensure there was no confusion in analyzing responses.
2. *Baseline:* The baseline was intended to ensure rooks performed at a consistent, stringently high level before manipulations. Commands were given as described above and as such, they contained not only the verbal auditory content of the command, but also potentially-subconscious eye gaze and other physical cues from the experimenter, natural vocal variation, pitch variation, and intonation variation. Proficiency at the Baseline ensures changes with further manipulations can be compared with a significantly-correct performance, but does not inform as to which part or parts of the signal are being used.

N.B. Only Leo received the following conditions, with the exception of one Memory Test, which was administered to all birds.

**Fig. 6:**
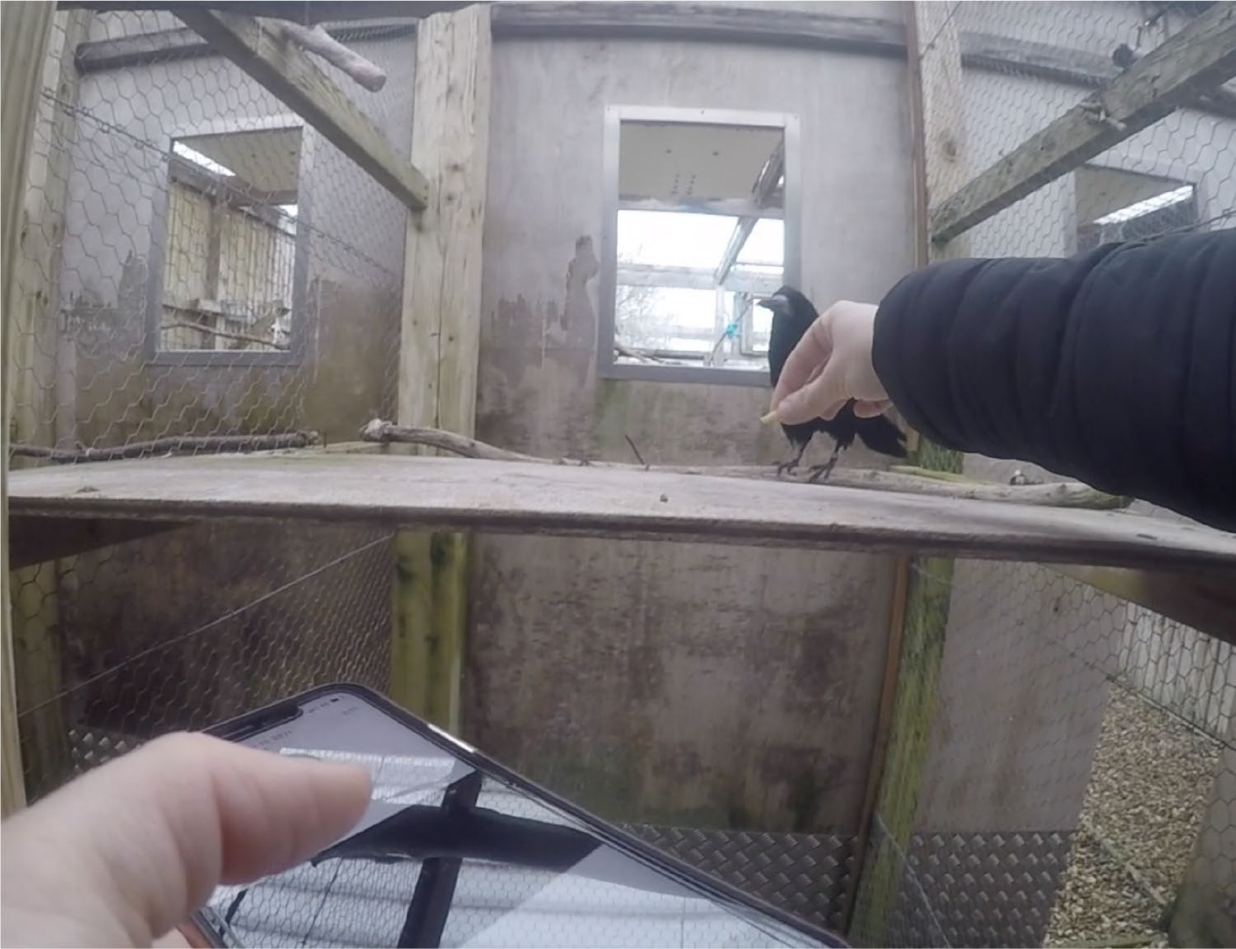
Leo, pictured, receiving trials for the recorded commands conditions, from the experimenter’s perspective

3. *Sunglasses*: Next, the experimenter performed commands as before, but obscured her eyes with non-reflective, dark sunglasses. Proficiency at this condition ensures no reliance on eye gaze cues (if proficiency is immediate) or the ability to compensate for the absence of these cues (if proficiency is acquired over time).
4. *Recorded commands*: Next, the experimenter performed commands as before, including the sunglasses, but commands had been previously recorded and were played through the speakers of an iPhone X. While placing the worm down on the table, the experimenter pressed play on the pre-determined command, and then responded to the birds’ behaviour as before (see Fig. 6). In this case, the experimenter continued to mouth the command by moving her lips as if giving the command; this was done with the goal of taking an additive approach of removing cues sequentially when possible. Proficiency at this condition ensures rapid generalisation to responding to commands from a recorded track, including fewer auditory components (if proficiency is immediate; see Fukuzawa et al. 2005a; Mills 2005), or the ability to compensate for the absence of these cues (if proficiency is acquired over time).

5. *Recorded commands Without Mouthing:* Next, the experimenter performed commands as in the previous condition, but ceased mouthing along to the commands. Proficiency at this condition ensures lack of reliance on the facial and lip movements associated with speech (if proficiency is immediate) or the ability to compensate for the absence of these cues (if proficiency is acquired over time).
6. *Partially-blinded condition*: Next, the experimenter performed commands as in the previous condition, but no longer knew which command was about to play from the recording. In order to accomplish this, audio tracks were given numbers from 1–60 as titles, and these numbers were randomised for each session. Of course, once the command begins playing the experimenter is no longer blind to the command, due to the need to reinforce a correct response. However, proficiency at this condition ensures lack of reliance on subconscious cues that may be given by the experimenter prior to command utterance and during the beginning of the utterance (if proficiency is immediate), or the ability to compensate for the absence of these cues (if proficiency is acquired over time).
7. *Memory tests:* During the course of the study, in two instances the main experimenter was away from the rooks for periods of about 6 months while conducting unrelated research. For Leo, the first occurred in the middle of a condition swapping the average pitch of commands with that of another command (in a following experiment, in prep), and the second occurred in the middle of conditions testing the effects of the biological sex and language accent of command speakers (in prep.). In the first instance, animal care technicians subsequently reported occasionally rewarding Leo’s responses to the commands; in the second instance, they were explicitly instructed *not* to do so and not to use the command words around the rook aviary. Upon the main experimenters’ return, she gave Leo refreshers of the Sunglasses condition (in the first case) and of the Baseline condition (in the second case) to ensure he remembered the commands before continuing with the relevant testing, and to run a short second test of whether the presence of sunglasses affected his performance. Proficiency at these conditions suggests long-term memory for the commands (if proficiency is immediate) or the ability to be reminded of the commands rather than needing to re-learn them from scratch (if proficiency is acquired over time).
8. *Controls:* In order to avoid affecting Leo’s motivation to respond to the commands with the extreme controls required to ensure no subconscious cueing occurred, controls were conducted only at the end of testing, once all other related experiments had been conducted. Other conditions given in further experiments, not reported here and carried out after the conditions reported here (with the exception of the Memory Tests, whose timing is described above), include, in order: flattening the pitch of each command to its average pitch, swapping the flattened average pitch of each command with that of another, examining the effects of biological sex and language accent of the command givers by utilising all combinations of male and female voices with British and American accents, assessing phonemic-like changes to each command, and switching to a new live experimenter (WML) (in prep.). After these conditions, to carry out controls, Leo received a set of Fully Blind controls, a set of Silent Controls, and then a set of normal Confirmatory Controls (using the original FMC recordings from the Recording condition):
  - *Fully blind:* To achieve this, the main experimenter (FMC) wore earbuds playing loud music while playing recorded commands from the iPhone X, with tracks identified by the number only. As in all recording trials, the experimenter would present the worm and then press play for the corresponding recording, but in this case the experimenter could not hear what command had played. The secondary experimenter, WML, stood off to the side of the main experimenter, mostly visually obstructed from the bird behind a wooden post and mesh, and indicated with a hand to the experimenter whether Leo should be given the worm, should be stopped from approaching, or should be allowed to retrieve the worm, depending on the correspondence of the command and behaviour. Although Leo had briefly experienced commands from WML before (in prep.), it is unlikely that subconscious visual cues from WML, in a non-presenting context, and who was partially visually obstructed, would account for successful performance. Additionally, Leo only experienced four rounds of commands from WML (in prep.). Leo should correctly respond to the commands here, despite the main experimenter being blinded, if he was indeed listening to the auditory component of the commands to make a choice.
  - *Silent:* On the other hand, if Leo was paying attention to auditory cues only, he should not be able to correctly choose a response if he could hear no command. To accomplish this, the main experimenter gave recorded commands as before, but this time the iPhone was connected to her earbuds. While she could hear the command being played and behaved accordingly during command presentation, Leo heard nothing and had to “guess” what to do based on the experimenter’s body language only.
  - *Confirmatory*: Normal recorded commands (as in the Partially-Blind condition) were given after these controls to ensure any loss of performance noted was not due to intrinsic motivational factors Leo was experiencing that day, but rather due to the manipulations of the controls themselves. All controls were carried out on the same day, in short sequence (although Leo left and returned a handful of times during testing, as was normal).

In each control, only 5 trials of each command type were given, forming a singular short round. The setup was complicated to administer for the experimenters, and likely aversive for the bird (particularly the Silent control). In similar controls for object permanence tasks, when birds had no information supplied, they often left and sometimes became reluctant to return (Cornero & Clayton 2025). It was decided not to jeopardise Leo’s willingness to participate in case further testing should ever be desired, so few control trials were offered. Accordingly, Leo was also intermittently given a worm randomly after some Silent control responses to ensure continued motivation, regardless of whether his response was correct or not. In controls, no sunglasses were used to ensure that maximum subconscious physical cues were available, and for conclusive evidence of success should Leo overcome them. Should Leo perform above chance in the Fully Blind Control, at or below chance in the Silent Control, and above chance in the Confirmatory Control, it would be strong evidence that he was listening to the auditory components of the commands and could compensate for lack of non-auditory signals.

### Statistical methods

Because only Leo was tested for long enough to pass the Baseline condition, birds’ performances were analysed individually, and results for Connelly and Fry can be found in the *Supplementary Materials*. When parametric measures were used, Box-Cox transformations were used to approximate normality in the analysed data: this transformation selects the exponent (lambda, λ) that best approximates a given dataset to normality (Box & Cox 1964; Sakia 1992). Should a log transformation be the best approach, the Box-Cox technique would suggest it as the best lambda (Box & Cox 1964). This was done because in all cases the data was non-normal and appropriate, paired non-parametric methods could not be used due to varying numbers of rounds conducted for each condition. All lambda values are reported, and residuals of all reported models were visually inspected via histograms and Q-Q plots after being fitted. ANOVAS were used after Box-Cox transformations to assess the effect of the experimental condition, the command type, how many rounds had occurred (for evidence of learning), and their interactions on the number of correct responses. Additionally, when possible, Leo’s last 5 sessions of one condition were compared with the first 5 sessions of the next condition with Wilcoxon signed-rank tests, to assess whether the manipulation introduced significantly affected his performance (see *Leo – Results: Performance Changes with Changing Conditions*). Leo’s Controls were compared to chance levels with binomial tests (chance = 0.5). Finally, linear regressions were fitted to each bird’s Baseline condition to assess learning rates.

## Results

**Fig. 7:**
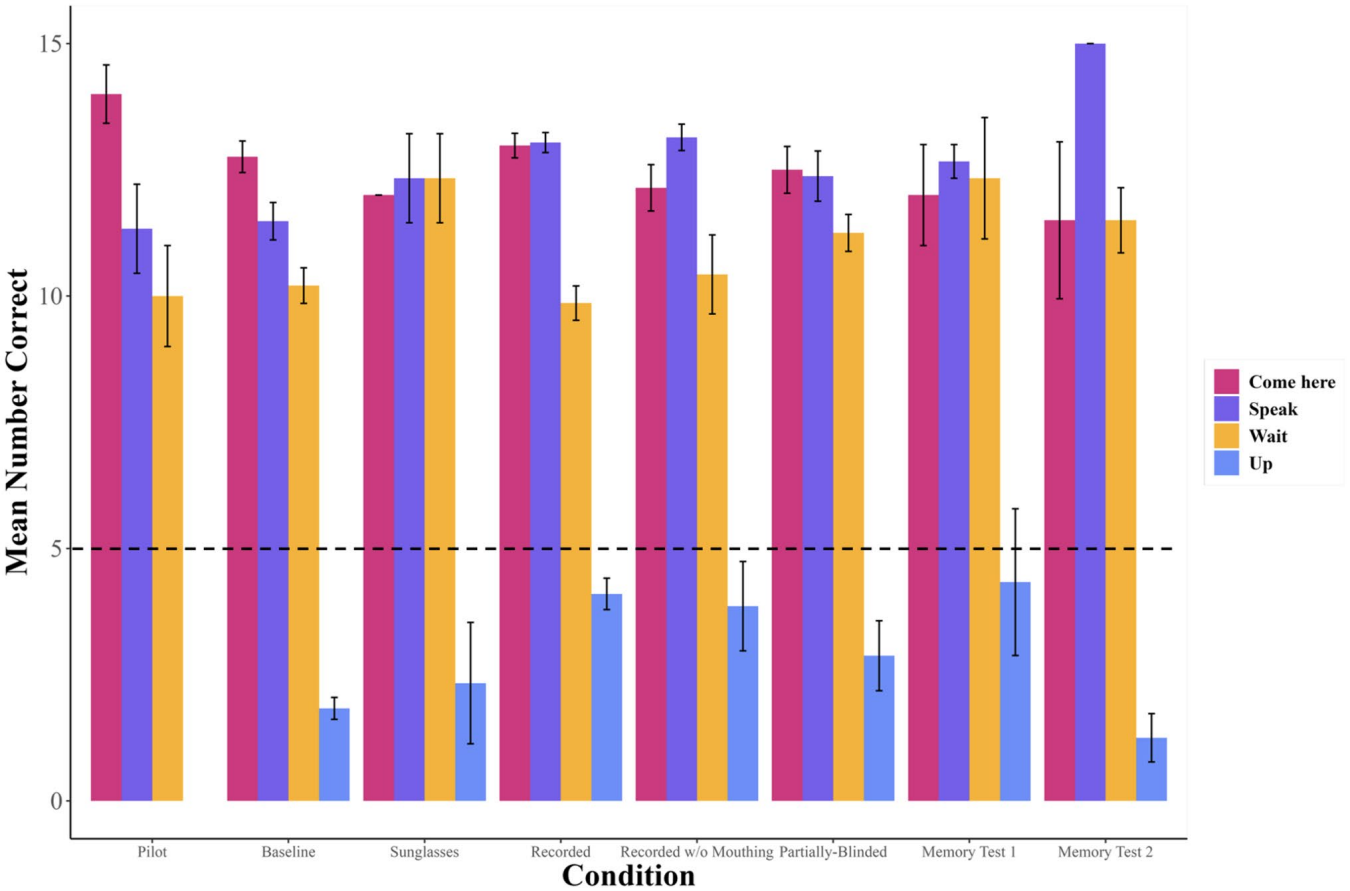
Leo’s overall average number of correct responses for each command, for each condition experienced. Conditions are listed in order given, but note that the Memory Tests did not occur immediately after the Partially-Blinded condition, nor did they occur immediately after one another (see *Methods*). The black dotted line represents chance at 1/3

During the time available for testing, only Leo passed the Baseline condition and was tested on all other conditions (note Fry and Connelly began command training about two years after Leo, and were tested for a much shorter period of time; taking all experimenter absences into account, they received about six months of actual testing time, compared to Leo’s almost two years of total testing time). Leo eventually reached criterion in all conditions (see Fig. 7), and his results are summarised in Table 1.

**Table 1:**
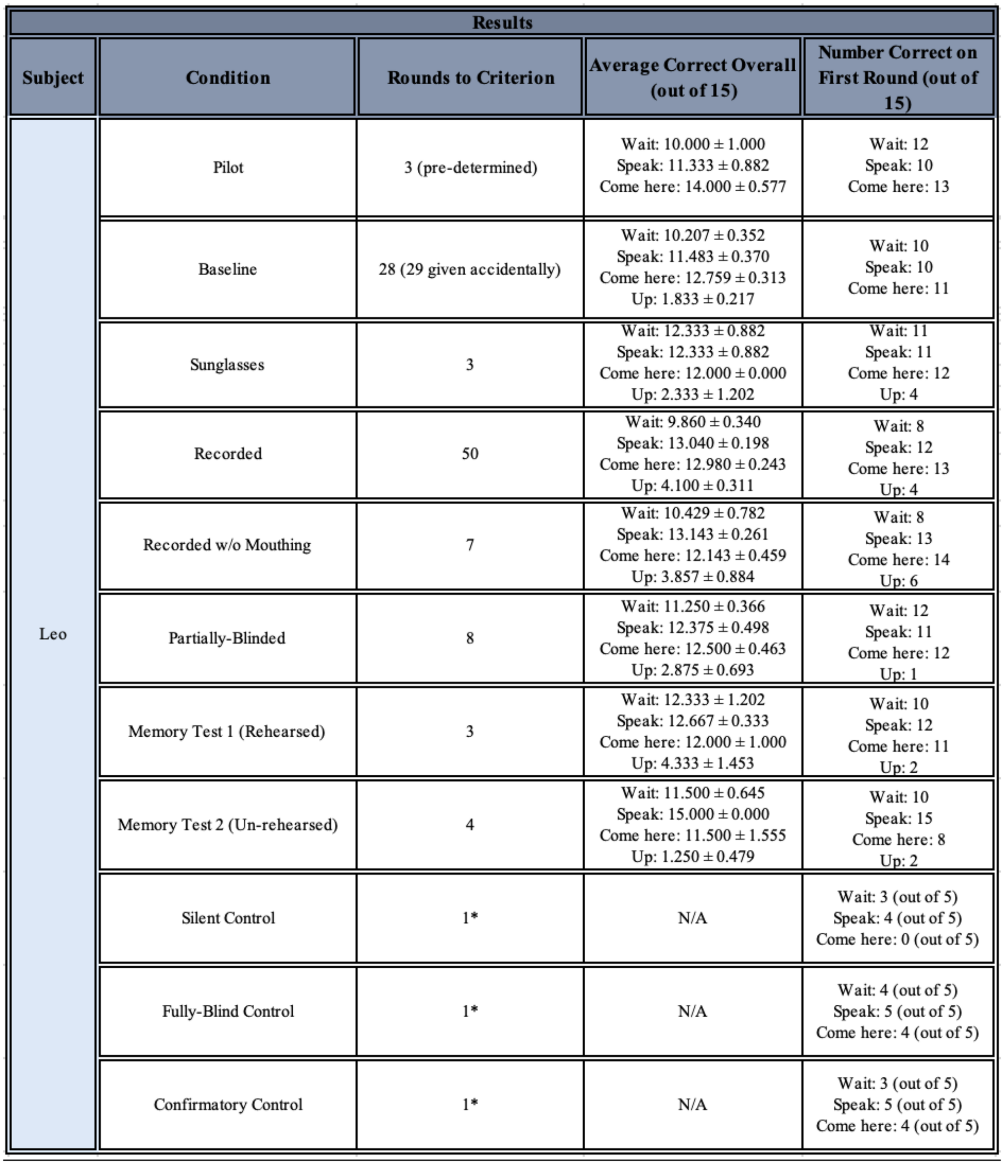
Leo’s results for each condition given, including number of rounds required to pass (or received, when applicable), overall average of correct responses per command, and number of correct responses per command on the first round received. Please note he received a single round of 5 of each command for each control

### Leo

*Pilot:* Leo received a Pilot condition of pre-determined length (3 rounds) before Baseline testing began, in which the experimenter assessed the methodology and whether behavioural criteria were sufficiently distinguishable. He had not yet achieved the required criterion for testing and continued to the Baseline to see how long it would be required for him to reach it.

*Baseline:* Leo required 28 rounds of Baseline command trials to reach criterion. An additional round was mistakenly given when this was not calculated correctly, but he remained above criterion at the end of Round 29. It could be concluded that Leo had learned the three main commands and performed them significantly correctly by the end of the Baseline set.

*Sunglasses:* Leo only required the minimum of 3 rounds to reach criterion for all three main commands when the experimenter donned non-reflective, opaque sunglasses. The addition of sunglasses was therefore not likely to have affected his ability to understand the command signal (more later, see *Leo – Overall Performance*).

*Recorded commands:* Leo required 50 rounds to achieve criterion on the three main commands when these were pre-recorded and played from an iPhone X’s speakers and the experimenter continued to silently mouth along to commands. The switch to a recorded medium, in which commands were in playback, was therefore likely to have affected his ability to understand or obey to the command signal, particularly for “Wait!” (more later, see *Leo – Overall Performance*). However, he was eventually able to overcome this effect, reaching criterion.

*Recorded commands without mouthing*: Leo required 7 rounds to achieve criterion on the three main commands when these were pre-recorded and played from an iPhone X’s speakers but the experimenter no longer lip-synced along to the commands. The removal of the lip movement associated with the commands was therefore likely to have affected his ability to understand or obey to the command signal somewhat, but apparently only for “Wait!” (more later, see *Leo – Overall Performance*): he rarely fell below criterion on “Speak” and “Come here”, although on three occasions he was 11/15 correct for “Come here”.

*Partially-blinded*: Leo required 8 rounds to achieve criterion on the three main commands when these were pre-recorded and played from an iPhone X’s speakers, but the experimenter no longer knew which command was about to play. The partial blinding of the experimenter to the commands may have affected his ability to understand or obey to the command signal somewhat, particularly for “Wait!”, but he also fell below criterion for “Speak” on occasion (more later, see *Leo – Overall Performance*).

*Memory test 1*: When Leo was tested in the same manner as the Sunglasses condition after 6 months without dedicated, daily command testing, but in which he had received a small number of informal command trials from animal care technicians (N.B., they did not know how the experimenter conducted trials, and instead used the same command words and accepted some unknown version of the behaviour to hand him a treat), Leo only took the minimum of 3 rounds to reach criterion on the three main commands. His memory for them after a period of 6 months with very little experience was remarkable.

*Memory test 2*: When Leo was tested in the same manner as the Baseline condition after 6 months without dedicated, daily command testing, and in which the technicians were instructed *not* to use any of the command words around him, Leo only took 4 rounds to reach criterion on the three main commands. Although by this point Leo had received extensive command experience over two years, his memory for the correct responses after 6 months without any refreshers was again remarkable. Additionally, his slightly worse performance without the Sunglasses, compared with the first Memory Test in which they were used, again suggests the addition of sunglasses did not significantly worsen his performance.

*Controls:* When Leo could hear the recorded commands played from the iPhone X’s speakers, but the experimenter administering the commands could not (Fully Blind Controls), he could still respond to the commands appropriately: he was correct on 13/15 commands given (binomial test, p = 0.007, two-tailed). He erred once on “Come here” and once on “Wait!”. On the other hand, when the main experimenter could hear the commands through earbuds and behaved accordingly, but no sounds played from the speakers for Leo to hear (Silent Controls), he was not able to tell what command he should perform: he was 7/15 correct on the commands given (binomial test, p = 1, two-tailed). Of note, he was correct on 3 instances of “Wait!” and 4 of “Speak”, but he never performed “Come here” at the correct time. This is not surprising because his behaviours were intended to be mutually exclusive, so one response was always considered to have occurred, and when the worm sat on the table but no command was given, Leo understandably would either attempt something (“Speak”) or wait for instruction (“Wait!”), which occasionally coincided with the command played. However, he never correctly performed “Come here”, despite his general high accuracy at that command in particular, further suggesting he could not hear the commands or read the experimenter’s body language, and that accuracy on “Speak” and “Wait!” was a side effect of his confusion. When commands were once again given as normal by the recording (Confirmatory Control), after these two controls, he was 12/15 correct on the commands given (binomial test, p = 0.035, two-tailed), indicating loss of performance in the second control was not external to the task design. He erred twice on “Wait!” and once on “Come here”. Together, these controls strongly suggest that, at least by the end of all command experiments, Leo was not being subconsciously cued by the experimenter to respond correctly, but rather had to rely on the auditory signal from the recording to choose behaviours significantly accurately.

### Overall performance

In order to examine the factors affecting Leo’s accuracy on the commands, after a Box-Cox transformation on the data, an ANOVA assessing the effect of the condition, the command type, the progression of time in number of rounds, and their interactions was carried out. There was no significant effect of condition, the interaction of condition and number of rounds, or the interaction of command type and number of rounds. However, there was a significant effect of command type, the progression of time in number of rounds, and the interaction of condition and command type (see Table 2). A post-hoc Tukey HSD test on the effect of the command type detected significant differences between “Wait!” and “Come here", and “Wait!” and “Speak”, but not between “Come here” and “Speak” (see Table 3). A post-hoc Tukey HSD test on the effect of the interaction between command type and condition detected a few significant differences of interest, excluding those for which categories were completely mismatched (i.e., comparisons between “Wait!” in the Baseline and “Come here” in the Sunglasses conditions were not considered to be meaningful, see Table 4). A post-hoc linear mixed-effects model on the effect of the progression of rounds found a significant increase in number of correct choices as rounds increased (0.392 ± 0.092, t = 4.250, p < 0.001).

**Table 2:**
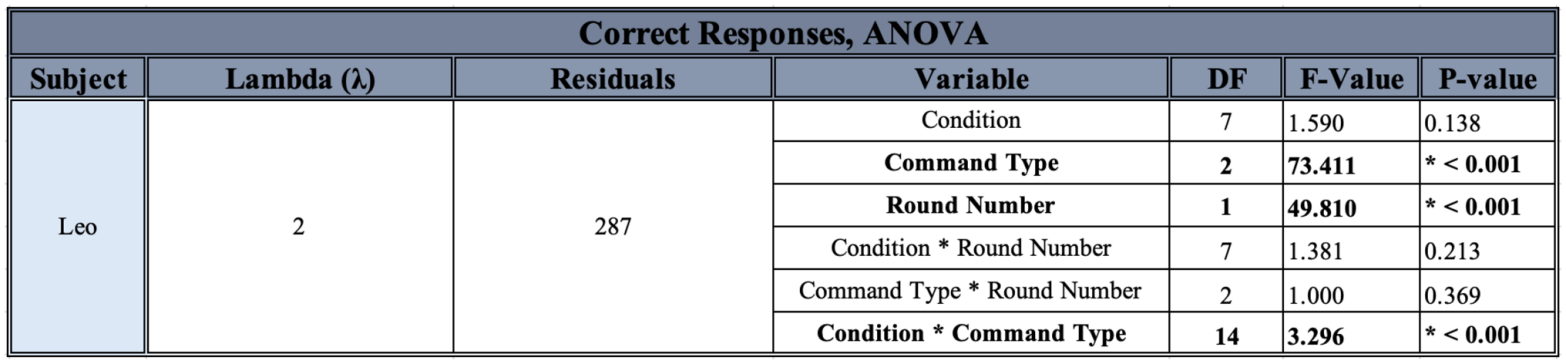
ANOVA output for the effect of various variables of interest on Leo’s performance. Significant results are denoted by an asterisk and bold lettering

**Table 3:**
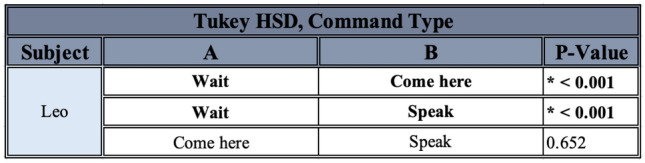
Post-hoc Tukey HSD test results for the effect of command type on Leo’s performance. Significant results are denoted by an asterisk and bold lettering

**Table 4:**
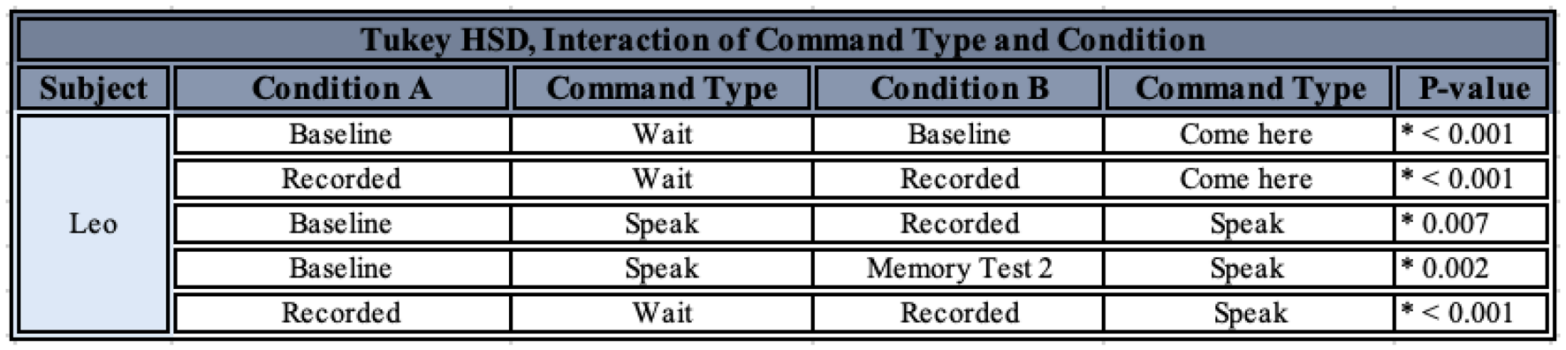
Post-hoc Tukey HSD test results for the effect of the interaction of command type and condition on Leo’s performance. Only significant results of interest (those excluding completely mismatched categories) are listed

**Fig. 8:**
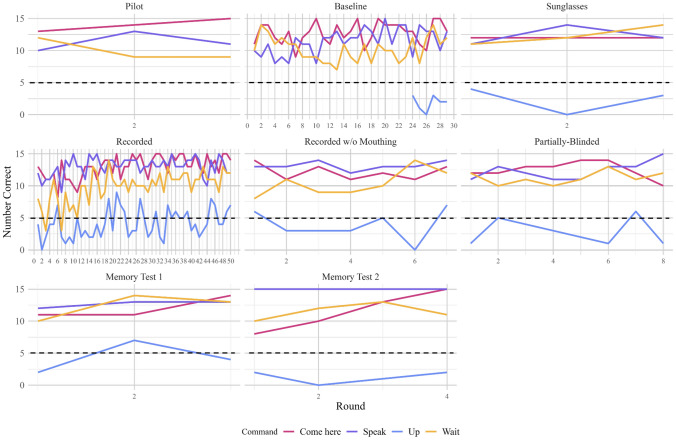
Leo’s number of correct responses for each command, for each round given and each condition experienced. Conditions are listed in order given, but note that the Memory Tests did not occur immediately after the Partially-Blinded condition, nor did they occur immediately after one another (see *Methods*). The black dotted line represents chance at 1/3

Leo displayed no major significant differences in his performance between different conditions, which is to be expected since he was always forced to return to the same criterion before moving on. However, he was significantly worse at correctly performing “Wait!” than both “Speak” and “Come here” throughout the experiment, whereas he was not more likely to be correct between “Speak” and “Come here”. He also generally improved in his performance as rounds progressed, which is again to be expected due to the requirement that he achieve criterion before moving on (see Fig. 7). There were also a few significant effects of note: he was significantly worse at “Wait!” than “Come here”, specifically for both the Baseline and the Recorded conditions, and worse at “Wait!” than “Speak” specifically for the Recorded condition. He was also better at “Speak” in the Recorded than the Baseline condition, but worse in the Recorded condition than in Memory Test 2 (see Fig. 8).

### Performance changes with changing conditions

**Fig. 9:**
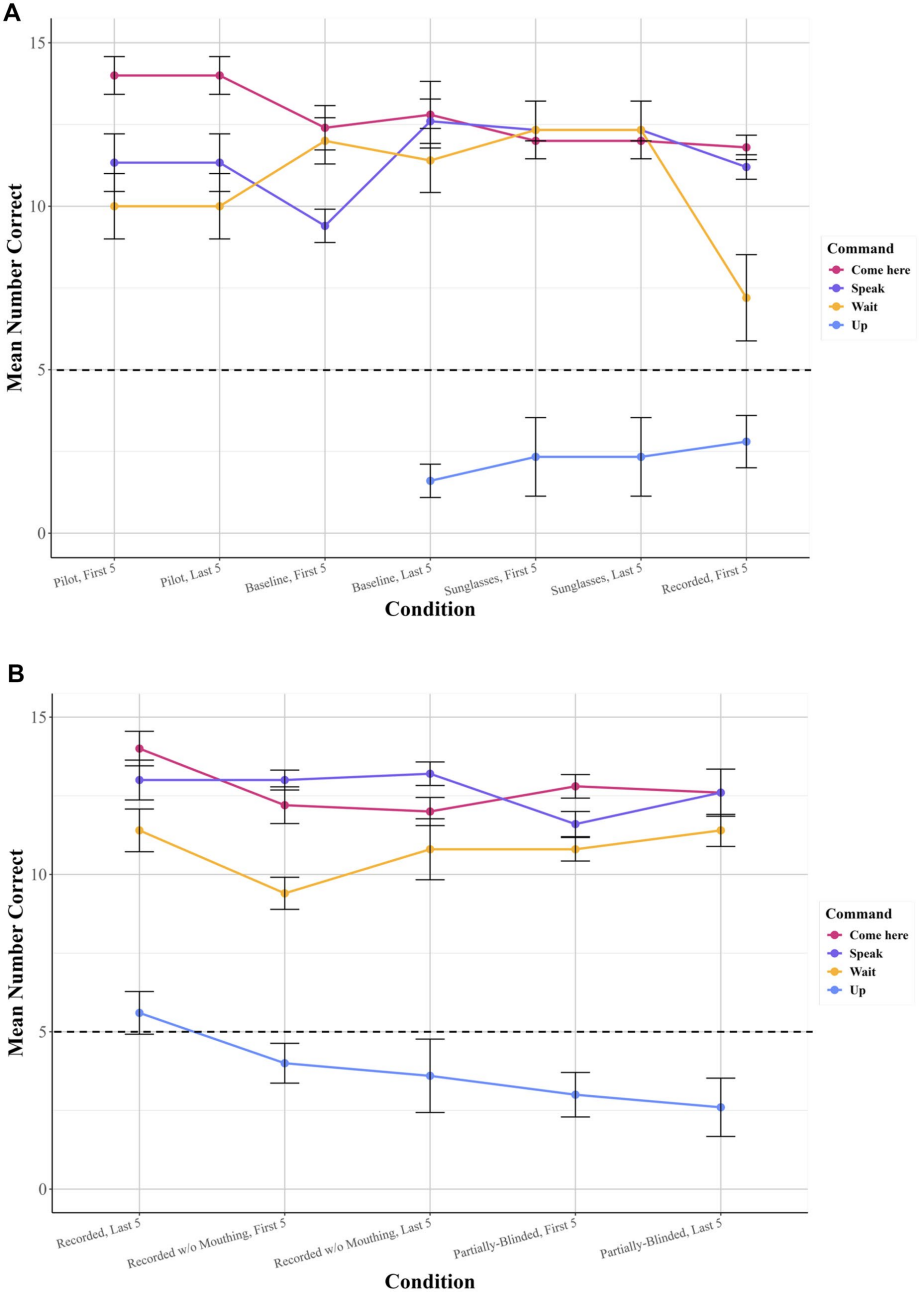
(**A**) Leo’s average number of correct responses for each command, for each condition experienced, with the first 5 rounds of each condition being juxtaposed with the last 5 rounds of the previous condition, for the Pilot, Baseline, Sunglasses, and Recorded conditions. Conditions are listed in order given, but note that not all conditions experienced 5 rounds: if less than 5 rounds were given, the average of all rounds given is used here (see *Results*). The black dotted line represents chance at 1/3 (**B**) Leo’s average number of correct responses for each command, for each condition experienced, with the first 5 rounds of each condition being juxtaposed with the last 5 rounds of the previous condition, for the Recorded, Recorded Without Mouthing, and Partially-Blinded conditions. Conditions are listed in order given, but note that not all conditions experienced 5 rounds: if less than 5 rounds were given, the average of all rounds given is used here (see *Results*). The black dotted line represents chance at 1/3

In order to examine any possible significant effect of changing conditions on Leo’s performance without the bias from the requirement to return to criterion each time, Leo’s performance in the last five rounds of some conditions were compared to his performance in the first five rounds of other conditions, to test for significant decrements in his performance after certain changes (see Fig. 9*, *Table 5).

**Table 5:**
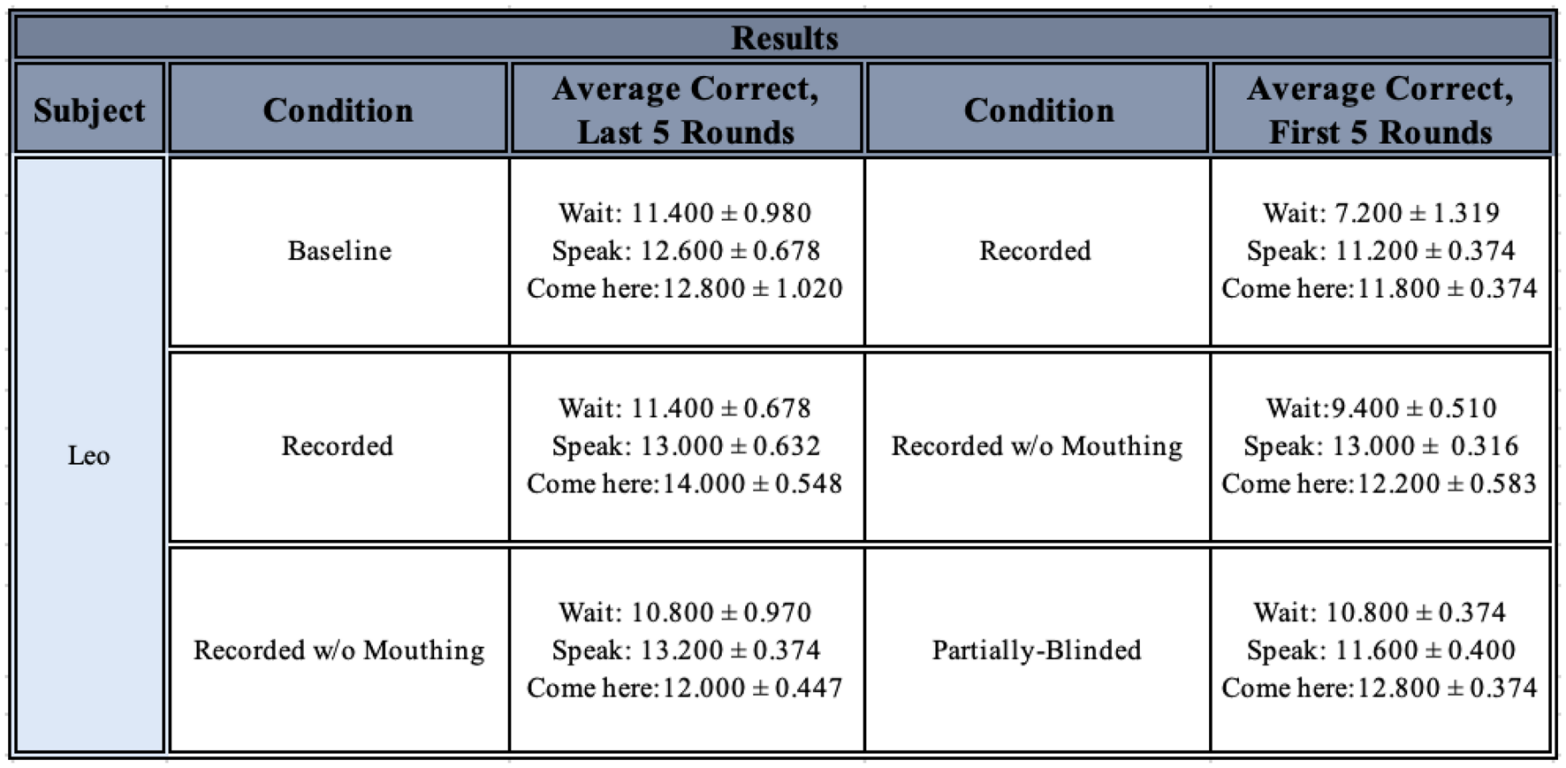
Average number of correct responses, for Leo, for the last 5 rounds of one condition and the first five rounds of the next condition, for comparison. Number correct is out of 15 possible attempts

The Baseline condition cannot be statistically compared with the Sunglasses condition because he passed the latter in only 3 rounds, and even non-parametric tests for small sample sizes (in this case, paired Wilcoxon signed-rank tests) require a larger sample size (for this test, n = 5).^2^ However, his passing within the minimum number of rounds required strongly suggests that the addition of sunglasses did not impair his performance. Accordingly, although the Baseline and Recorded conditions were not carried out one after the other (the Sunglasses condition was in between), the short lapse between them (and the lack of the effect of the addition of Sunglasses on his performance) allows for the last 5 rounds of the Baseline to be compared to the first 5 rounds of the Recorded conditions without much loss in conceptual validity. There was a significant difference: Leo’s performance on the first few rounds of the Recorded condition was worse than his passing performance by the end of the Baseline (Wilcoxon signed-rank test, V = 15.5, p = 0.021). It seems likely, corroborated by the high number of sessions required for Leo to pass in the Recorded condition (50, higher than any other condition tested here or in subsequent related experiments, in prep.), that the switch from live audio to a recording significantly affected Leo’s performance.

The Recorded and the Recorded Without Mouthing conditions were conducted one after the other, and so can be compared easily. There were significant differences (Wilcoxon signed-rank test, V = 10, p = 0.024). As such, despite passing the Recorded Without Mouthing condition much faster (in 7 rounds), it does appear that the removal of the concordant lip movements initially significantly affected Leo’s performance. Plausibly, however, he recovered from this effect relatively quickly, and this appeared to be the case only for “Wait!”.

The Recorded Without Mouthing and the Partially-Blinded conditions were conducted one after the other, and so can be compared easily. No significant differences were found (Wilcoxon signed-rank test, V = 53.5, p = 0.727). As such, although he took a similar amount of rounds to pass both conditions (relatively more than the Sunglasses condition, but much less than the Recorded condition), it does not appear that the blinding of the experimenter to the command that was going to play next influenced Leo. In other words, he was unlikely to be strongly relying on subconscious experimenter cues to determine what behaviour he had to perform, and he was able to compensate for the change in condition rapidly.

The Memory Tests occurred out of sequence with these other conditions; additionally, he took less than 5 rounds to pass each. As such, they cannot be compared to other conditions in this manner. Furthermore, since it is not logical to compare changes in performance between rounds of trials carried out very distantly from one another (as any number of other factors could account for his change in performance, at very different temporal periods, in addition to the changes implemented), no other conditions were compared against each other in this manner.

## Discussion

Rooks, an undomesticated corvid songbird species, can learn to follow human communicative cues in the form of verbal vocal commands. One rook, Leo, learned three vocal commands sufficiently well to reach a strict criterion of proficiency, and two more rooks, Connelly and Fry, showed that they were likewise learning the commands (but likely did not have enough time to reach proficiency; see *Supplementary Materials*). In addition, by the end of the experiment Leo was using the auditory components of the commands themselves to decide which behaviour to perform, as demonstrated by sequential removal of several cues that Leo could have used to correctly respond to the commands (including eye gaze, a complete and full auditory signal in the form of live speech, lip movements, and any potential subconscious cueing by the experimenter). Two memory tests, conducted each time after about 6 months without extensive command training, and in one of these cases in absence of any rehearsal, showed that Leo retained a long-term memory for the commands and their correct responses. Taken together, these results suggest that functional, highly accurate utilisation of human communicative speech sounds need not be restricted to either domesticated species or highly enculturated individuals, and provide documented evidence of command-learning and the cues used in an avian species, at least according to the performance of one individual. Rather, Leo’s performance suggests that other cognitively and socially-complex species may be able to utilise cognitive and/or communicative abilities evolved for reasons unrelated to communicating with humans in order to functionally use human cues to achieve desired outcomes, such as obtaining food rewards. This may be particularly true for groups with pre-existing complex communication systems, such as corvids, which like other songbirds learn their songs.

When direct comparisons are possible, Leo behaved similarly to domestic dogs, the species for which the most dedicated command research is available. Like the dogs in Fukuzawa et al. 2005a, b studies, Leo had some unquantified prior experience with the commands, and had to achieve a similar criterion to pass onto further conditions (for the dogs, 17/20 correct on each of two commands in a single day; for the rooks, 12/15 correct on each of three commands, on average, over three consecutive sessions). He performed similarly to the dogs on similar conditions: like the dogs, Leo did not appear to have his performance significantly affected by the addition of sunglasses, and therefore was unlikely to be using gaze cues (Fukuzawa et al. 2005a). Leo and the dogs were both significantly affected by a switch from live to recorded commands, taking the highest number of rounds to re-establish competence (although note that the dogs were initially tested on recorded commands without sunglasses, and their performance suffered both then and when sunglasses were re-added, Fukuzawa et al. 2005a). While the effect of lip or other facial movements was not assessed in dogs, Leo’s performance suffered slightly when these movements were omitted, indicating that some element of this cue was previously used. The dogs were assessed for physical cues given by the experimenter (with the experimenter at different distances, sitting/standing, and levels of visual separation) in a manner not possible with Leo, with increasing experimenter distance and decreasing visual access to the experimenters affected the dogs’ performance (Fukuzawa et al. 2005a). Contrastingly, there is no strong evidence that Leo was using physical cues given by the experimenter intentionally or unintentionally, aside from an initial effect of lip movements: he did not suffer from a significant drop in performance when the experimenter was partially blind to the command which would play next, and he was significantly correct during a short set of control commands in which the experimenter was completely blinded to the command being played. Critically, Leo did not significantly succeed when the experimenter could hear the commands but Leo heard nothing.

Of additional note, the rooks’ set of commands was likely to be more difficult than those typically presented to dogs. In most studies assessing command obedience in dogs, there was no testing of a command such as “Wait!”, which requires the ability to delay gratification by avoiding approaching food during the entire delay period (in addition to initially choosing the correct behaviour out of the set). For the rooks’ “Wait!”, they had two manners to err: choosing the incorrect behaviour from the beginning (performing some other action), but also choosing the correct response (to begin waiting) but being unable to refrain from taking the worm until the experimenter allowed it. It is conceivable that this dichotomy may have caused more errors with this command, particularly in more difficult conditions, than any “one-time” command the dogs experienced in the most directly-comparable studies (i.e. Fukuzawa et al. 2005a, b; Mills et al. 2005; Sturdy et al. 2022). Here, correct performance at “Wait!” was significantly lower than for either “Speak” or “Come here” for all birds, and Leo’s performance decreases (when switching to a recorded command and when removing lip movements) appear primarily mediated by decreases in performance specifically for “Wait!”. All birds also achieved proficiency at the other commands (excluding “Up”, which was never learned) much sooner than “Wait!”. Leo’s competence might not have been significantly affected by any of the manipulations if only “Come here” and “Speak” had been studied here. It is possible that the use of a more-difficult command such as “Wait!”, which added an additional cognitive load in the form of inhibitory control or executive function, may have unearthed effects in Leo that otherwise might have been missed.

Although the “ease” of teaching these birds these commands is impossible to quantify, especially for Leo (whose prior experience was not recorded before some proficiency had been noted; see *Methods*), qualitatively, these rooks were remarkably easy to train (FMC, personal observations). Leo, as mentioned (see *Introduction* and *Methods*), appeared to learn “Come here” incidentally, simply from the experimenter using it during habituation, and he began performing “Speak” on cue within a few instances of receiving worms for spontaneously making a call after the command was uttered (i.e. in one “training” session). Fry behaved similarly; indeed, for “Speak” specifically, rooks appeared to “enjoy” this command or to be primed for it. In all cases, once they began performing the correct response, they would engage in “Speak” repeatedly and frequently, initially even in absence of any request (Leo, for a period of about a day, would follow the experimenter around the aviary while screaming). Additionally, although qualitatively rooks initially seemed to make full call sequences in response to “Speak” (including multiple utterances, in a stereotyped, predictable order), after some time they began shortening their calls, eventually performing a singular, low and guttural utterance instead (similar to what was noted for the crows also trained to make calls, Brecht et al. 2019). As is the case for crows, this suggests rooks have flexible and volitional control over their vocalisations for this purpose (Brecht et al. 2019; Liao et al. 2024). This may be another example of rooks intentionally modifying their calls' circumstances, as they appear to do when confronted with external auditory stimuli (Martin et al. 2025a), or it may be related to some other aspect of complex communication with conspecifics, which remains poorly understood despite some attempts to study it (i.e. Martin et al. 2022, 2024; 2025b).

Leo appeared to be attending primarily to the auditory components of the commands when choosing what behaviour to perform. A switch from live commands to a recording appeared to have the strongest effect on his performance, although these conditions did not allow for determination of what part of the *auditory* component(s) of the commands he was attending to when he heard the command sounds. These commands varied in their pitch, intonation, and change in pitch over the entire command, in order to be naturalistic; additionally, each command is made up of different phonetic components, and in this case, the voice of only one female experimenter was used. Further experiments assessing the effects of other components of the auditory characteristics of the commands (average pitch, pitch and tonal changes, the biological sex and accent of experimenters, switching to receiving commands from another live experimenter, and phonemic components of the command words) have been conducted and are currently in preparation for publication.

In sum, Leo’s performance contradicts hypotheses that processes incurred during domestication in some species (such as the dometic dog) are *necessary* for either a tendency to attend to human communicative cues, for an ability to learn to use such cues correctly and functionally, or both (Fukuzawa et al. 2005a; Gibson et al. 2014; Mills et al. 2005; Soproni et al. 2001; Sturdy et al. 2022). Should that have been the case, Leo should have been unable to learn these commands or should have struggled significantly to do so. Likewise, although these rooks were hand-raised and had some experimental experience, and were familiar with human experimenters and caretakers, they did not have extensive direct experience, at short distances, with human cues and interactions. The rooks lived in social groups with other rooks in large, outdoor aviaries, spending most of their time without human company. Most if not all, of their prior studies can be described as primarily observational, and they required long periods of extensive habituation before they were willing to participate in any experiments at close range with the main experimenter (see Cornero & Clayton 2025). Due to this, and given that these rooks were already adults at the time any commands were introduced, it is likely that extensive human enculturation from a young age is also not *necessary* for sensitivity to and functional use of human cues by nonhuman animals, at least not in all species. It is still possible that either of these factors (domestication, enculturation, or both) may be supportive of or facilitate such abilities in some species, but they are not *required* per se.

Instead, as others have suggested, it is likely that at least some species are able to hijack existing domain-general processes (such as those required for conspecific communication or supportive of rapid learning), or other cognitive abilities (such as complex social cognition and reasoning by exclusion), to attend to, learn, and use human communicative cues, including vocal speech (Andics et al. 2016; Kaminski et al. 2004; Kriengwatana et al. 2015). Owing to their complex cognition, complex and varied social systems, and complex communicative abilities, examining other corvid species for the ability to co-opt auditory human communicative signals is likely to be highly fruitful for future research. For instance, comparing corvid species with various degrees of sociality versus those with more solitary lifestyles, or those with greater levels of vocal complexity versus those for which calls are more stereotyped and limited, may begin to allow determination of which cognitive and communicative abilities are used for such goals, and provide information as to which neural systems work together and how they do so (e.g. Liao et al. 2025).

This study joins a small body of work demonstrating that undomesticated species, including sea lions, walruses, dolphins, cats, crows, macaws, and more can learn to attend to auditory human communicative cues and/or utilise human speech sounds effectively with regards to the following of human commands (Brecht et al. 2019; Endo et al. 2020; Herman et al. 1984; Liao et al. 2024; Saito et al. 2019; Sasaki et al. 2022; Torres Ortiz et al. 2022). It also complements a large body of work establishing and examining functional referential verbal communication with another cognitively-complex avian species, the African grey parrot. Importantly, the work on African grey parrots, unlike the present study, goes beyond basic training of an engrained response for a presented stimulus, and rather demonstrates that these birds have a functional understanding of label categories, higher-level relations, numerosity, referential comprehension, and more (e.g. Pepperberg 1990; 2012a,b; 2014). To the best of our knowledge, this is the first controlled experiment studying human vocal/verbal *command* comprehension and, particularly, the specific cues attended to in the process by a non-mammalian, non-domesticated species. Although the results presented here are primarily based on the performance of one very successful rook, further comparative research, including a greater number of dedicated command-based studies in a wider phylogenetic variety of species, should be undertaken. Key future directions include determining the underlying cognitive processes allowing some nonhuman species to co-opt heterospecific communicative cues in the form of responding to human vocal communication, and understanding which cues are attended to when responding to commands and how these vary by species.

## Supplementary Information

Below is the link to the electronic supplementary material.

Supplementary file1 (MOV 140768 KB)

Supplementary file2 (MOV 111939 KB)

Supplementary file3 (MOV 221555 KB)

Supplementary file4 (MOV 94942 KB)

Supplementary file5 (MOV 129201 KB)

Supplementary file6 (MOV 95861 KB)

Supplementary file7 (MOV 87875 KB)

Supplementary file8 (DOCX 3776 KB)

## Data Availability

The dataset used to analyse the data can be obtained at: (https://osf.io/vj9f5/?view_only=8fc62be88b0f41b9a0f9fd7f5778c0d9). Additional materials can be made available upon reasonable request.

## Footnotes

1 As discussed in Cornero & Clayton (2025) for object permanence tasks for the same sample of birds, chance was kept at 0.5 despite more than two possible responses being available (in this case, up to four possible behaviours, chance = 0.25) because of the impossibility of assigning probabilities to what actions a bird might choose to take. Therefore, the standard correct vs. incorrect probability of 0.5 is used here, which is highly conservative.

2 Wilcoxon signed rank tests statistical tables for small sample sizes do not allow for significance to be found when n < 5; e.g. Corder & Foreman (2014).

## Declarations

### Open practices

This experiment was pre-registered with the Open Science Framework prior to data collection. Methods and analyses described here are as ultimately conducted (when differing from pre-registration due to necessity). See (https://osf.io/vj9f5/?view_only=8fc62be88b0f41b9a0f9fd7f5778c0d9).
