# Supplementary material for: Can an old rook learn new tricks? Vocal command comprehension and obedience in rooks (Corvus frugilegus)
Source: Anim Cogn. 2025 Sep 9;28(1):81. doi: 10.1007/s10071-025-02002-8 (PMC12420768; doi:10.1007/s10071-025-02002-8)
Supplement: Supplementary file 8 — Supplementary file8 (DOCX 3776 KB) [file 10071_2025_2002_MOESM8_ESM.docx]

**Supplementary Materials**

*Additional Subjects, Methodological Changes:*

Fry and Connelly, who began command training much later and with the experiment in mind, were trained in the testing compartments from the beginning, with the same visual presentation for all commands, unlike Leo. Although initially they were also trained in a directed manner by FMC to perform the desired behaviours, once they reliably performed all three behaviours some of the time (but not necessarily in response to the correct command), proficiency testing began in the form of pseudorandomised sets of trials that were scored as their Baseline conditions. As such, Fry and Connelly likely began proficiency testing at a much lower level of proficiency than Leo, who only needed to transfer commands he appeared to already have learned. For a summary of each birds’ training and testing experiences, see *Supplementary Table 1*.

Because Leo never became proficient at “Up” over the course of the study, Fry and Connelly were not offered “Up”. Fry occasionally had a chance to observe Leo’s trials because she would wait in an adjacent compartment for her turn (given that she suffered from arthritis in one foot, the experimenter decided not to cause her pain by making her move unnecessarily); however, Leo would usually flush her away himself.

For Connelly, who was reluctant to approach and would not participate if the worm was too close to the experimenter, the reward worm was placed closer to the edge of the testing table. For Fry, it was placed at least ~1 foot away from her if she chose to remain on the testing surface (she suffered from arthritis in one foot, and it would not have been ethical to force her to move around unnecessarily to reset a trial).

For “Come here”: unlike Leo, other birds, who were much more hesitant to approach (Connelly, due to neophobia to the experimenter, see also Cornero & Clayton, 2025) or moved slowly (Fry, due to her arthritis), only had to begin physically moving towards the worm within the time frame to be considered correct.

For “Speak”: Connelly made a characteristic neck stretching and looking from side to side movement prior to opening the beak and making a sound, which took some time; if he began this set of movements before the time elapsed *and* made a sound as expected immediately after the conclusion of these movements, he was considered to be correct even if the sound itself did not occur within 3s. If he began this set of actions but did not ultimately make a sound immediately thereafter, he was considered to be incorrect.


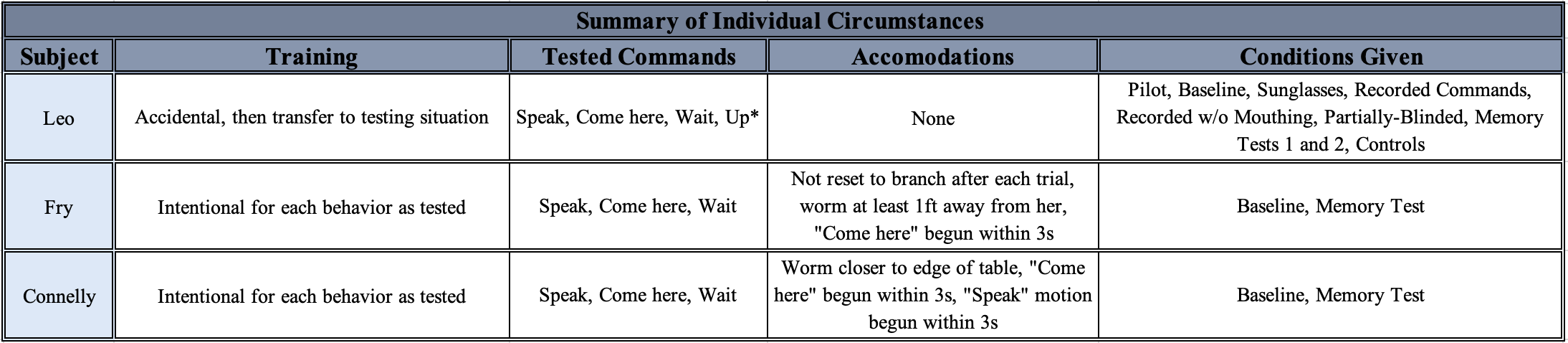


**Supplementary Table 1** Summary of each bird’s training and testing experiences, including commands given, any accomodations allowed, and conditions experienced. Note that “Up” was not scored for Leo, although it was given

*Additional Subjects, Results:*

Fry:

Fry worked slowly, being tested on only a few command trials per day, and accordingly received few rounds of Baseline testing before the experiment ceased. She also received a Memory Test when the experimenter had been absent for 6 months during her Baseline testing (see *Supplementary Figure 1*): unlike Leo’s Memory Test, this limited, 3 round test was not intended to assess whether she remembered commands that she had not yet sufficiently learned, but rather to give her a chance to refresh her memory before continuing with Baseline testing. Her results for this test are also reported here: it occurred after the period of time in which technicians were instructed *not* to use the command words in the rook aviary, nor to practice commands with the birds.


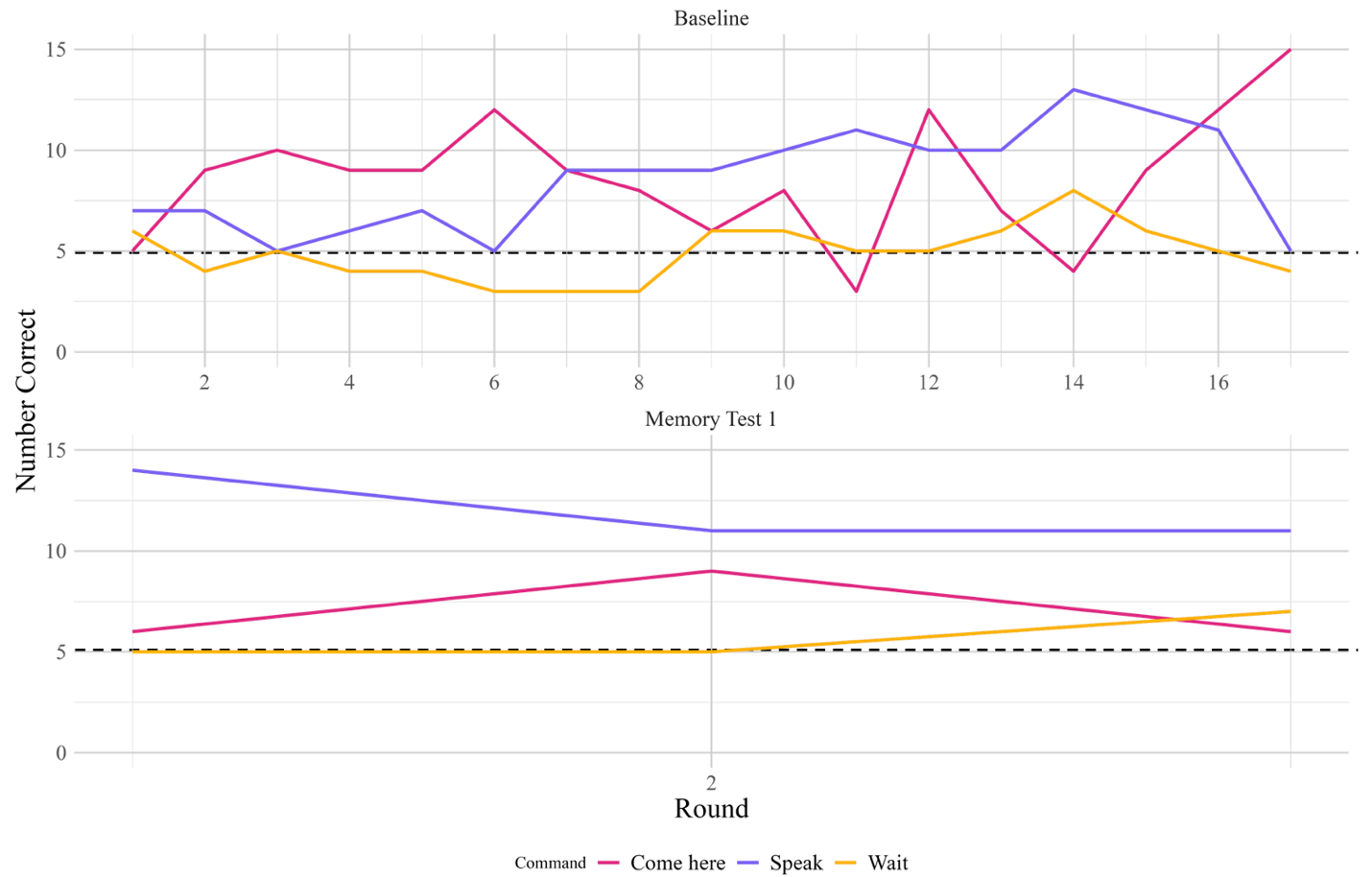


**Supplementary Fig. 1** Fry’s number of correct responses for each command, for each round given and each condition experienced. Note that her Memory Test occurred during her Baseline testing, and was unrehearsed (see *Results*). The black dotted line represents chance at 1/3

*Baseline*: Fry received 17 full rounds of Baseline testing only. She did not achieve criterion (see *Supplementary Figure 2, Supplementary Table 2*).

*Memory Test:* Fry also received a memory test after 6 months without commands, although note she had not achieved criterion yet (see *Supplementary Figure 2*, *Supplementary Table 3*).

**
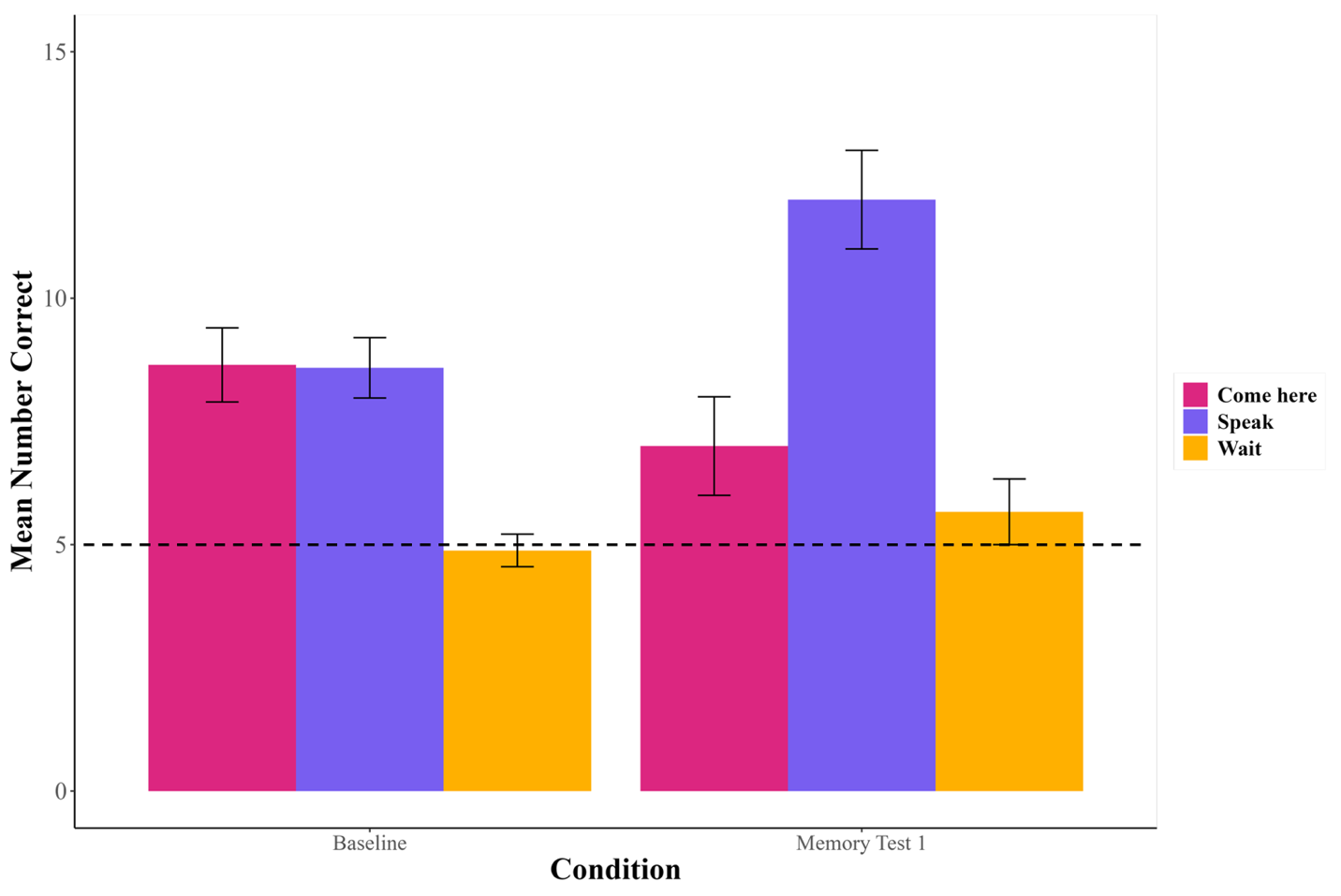
**

**Supplementary** **Fig. 2** Fry’s average number of correct responses for each command, for each condition experienced. Note that her Memory Test occurred during her Baseline testing, and was unrehearsed (see *Results*). The black dotted line represents chance at 1/3


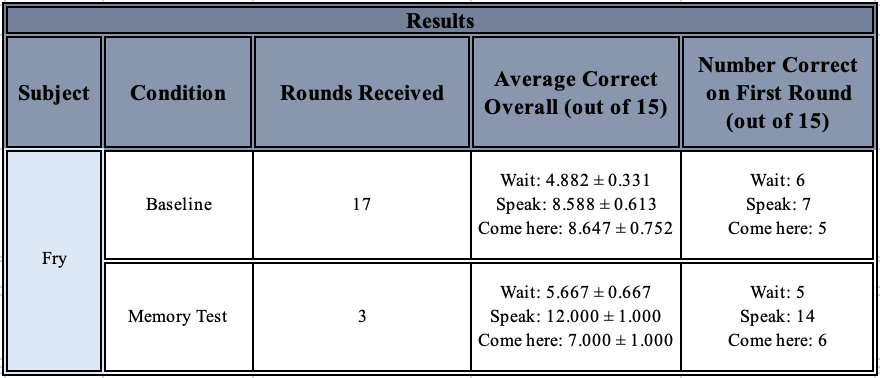


**Supplementary** **Table 2** Testing results for Fry, including rounds received for each condition, average correct responses overall, and number of correct responses on her first round of each condition. Note that Fry never achieved criterion

*Overall Performance:*

In order to examine the factors affecting Fry’s accuracy on the commands, after a Box-Cox transformation on the data, an ANOVA assessing the effect of the condition, the command type, the progression of time in number of rounds, and their interactions was carried out (*Supplementary Table 3*). There was no significant effect of condition, the interaction of condition and number of rounds, the interaction of command type and number of rounds, or the interaction of condition and command type. However, there was a significant effect of command type and of the progression of time in number of rounds. A post-hoc Tukey HSD test on the effect of the command type detected significant differences between “Wait!” and “Come here" and “Wait!” and “Speak”, but not between “Come here” and “Speak” (see *Supplementary Table 4*). Unlike the ANOVA, a post-hoc linear mixed-effects model on the effect of the progression of rounds found no significant increase in number of correct choices for increasing rounds, but the trend was positive (0.019±0.017, t = 1.131, p = 0.263). Therefore, although Fry did not learn the commands in the time allotted, there was a trend suggesting that she was learning them over time, and “Wait!” generated a poorer performance than the other commands, as for Leo.


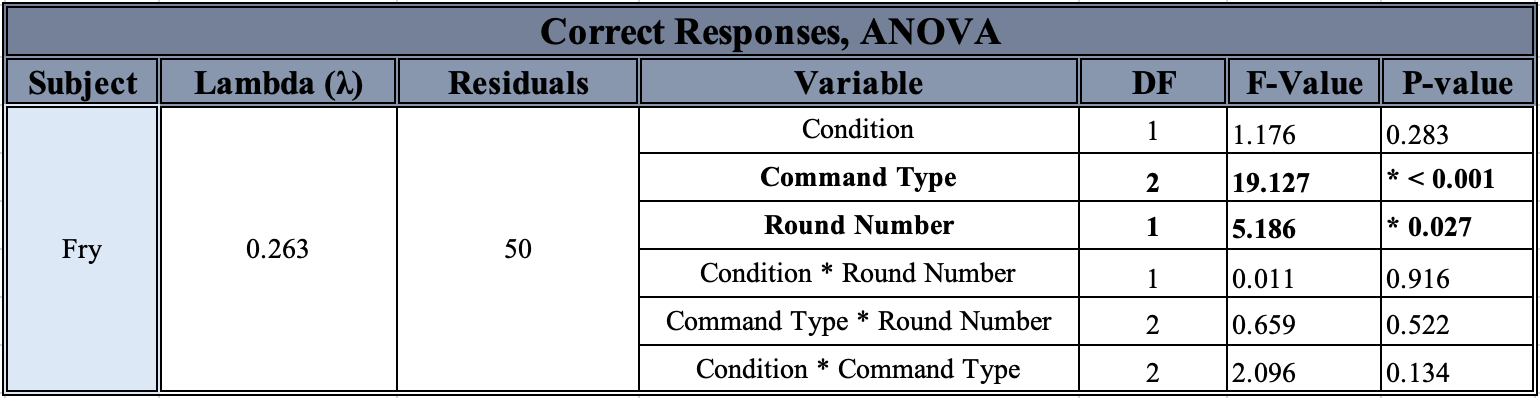


**Supplementary** **Table 3** ANOVA output for the effect of various variables of interest on Fry’s performance. Significant results are denoted by an asterisk and bold lettering


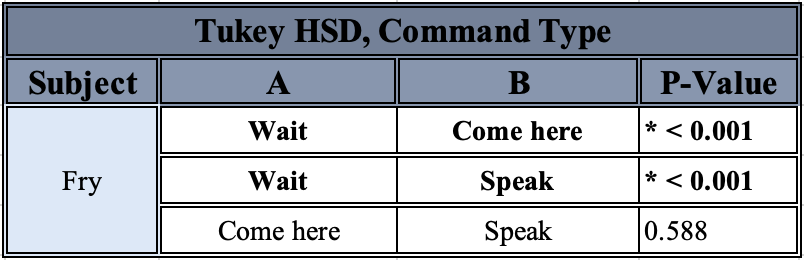


**Supplementary** **Table 4** Post-hoc Tukey HSD results for the effect of command type on Fry’s performance. Significant results are denoted by an asterisk and bold lettering

Connelly:

Connelly worked more rapidly than Fry, and as such was able to complete many more rounds of Baseline testing before the experiment had to cease. He also received a Memory Test when the experimenter had been absent for 6 months during his Baseline testing (see *Supplementary Figure 3*); unlike Leo’s Memory Test, this limited, 3 round test was not intended to assess whether he remembered commands that he had not yet sufficiently learned, but rather to give him a chance to refresh his memory somewhat before continuing with Baseline testing. His results for this test are also reported here, and it also occurred without him receiving any command practice during the 6 months in which he was not tested, like Fry and like Leo’s Memory Test 2.

**
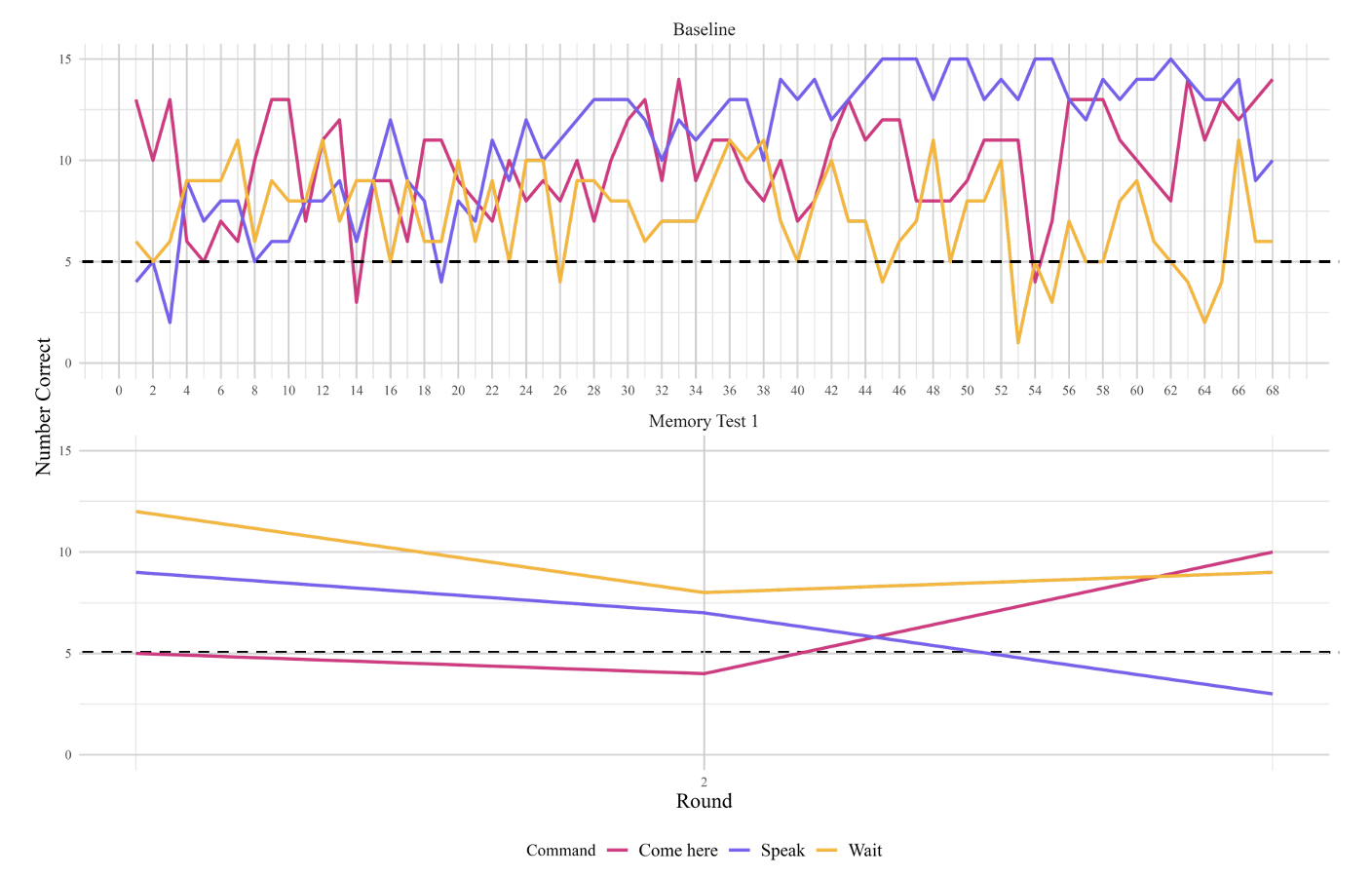
**

**Supplementary** **Fig. 3** Connelly’s number of correct responses for each command, for each round given and each condition experienced. Note that his Memory Test occurred during his Baseline testing, and was unrehearsed (see *Results*). The black dotted line represents chance at 1/3

*Baseline*: Connelly received 68 full rounds of Baseline testing. He did not reach criterion during this time (see *Supplementary* *Figure 4, Supplementary* *Table 5*).

*Memory Test:* Connelly also received a memory test after 6 months without commands, but note he had not reached criterion at that point (see *Supplementary* *Figure 4, Supplementary* *Table 5*).


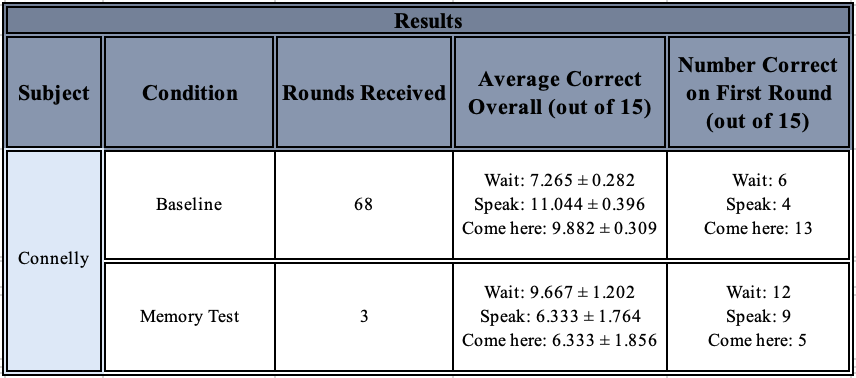


**Supplementary Table 5** Testing results for Connelly, including rounds received for each condition, average correct responses overall, and number of correct responses on her first round of each condition. Note that Connelly never achieved criterion


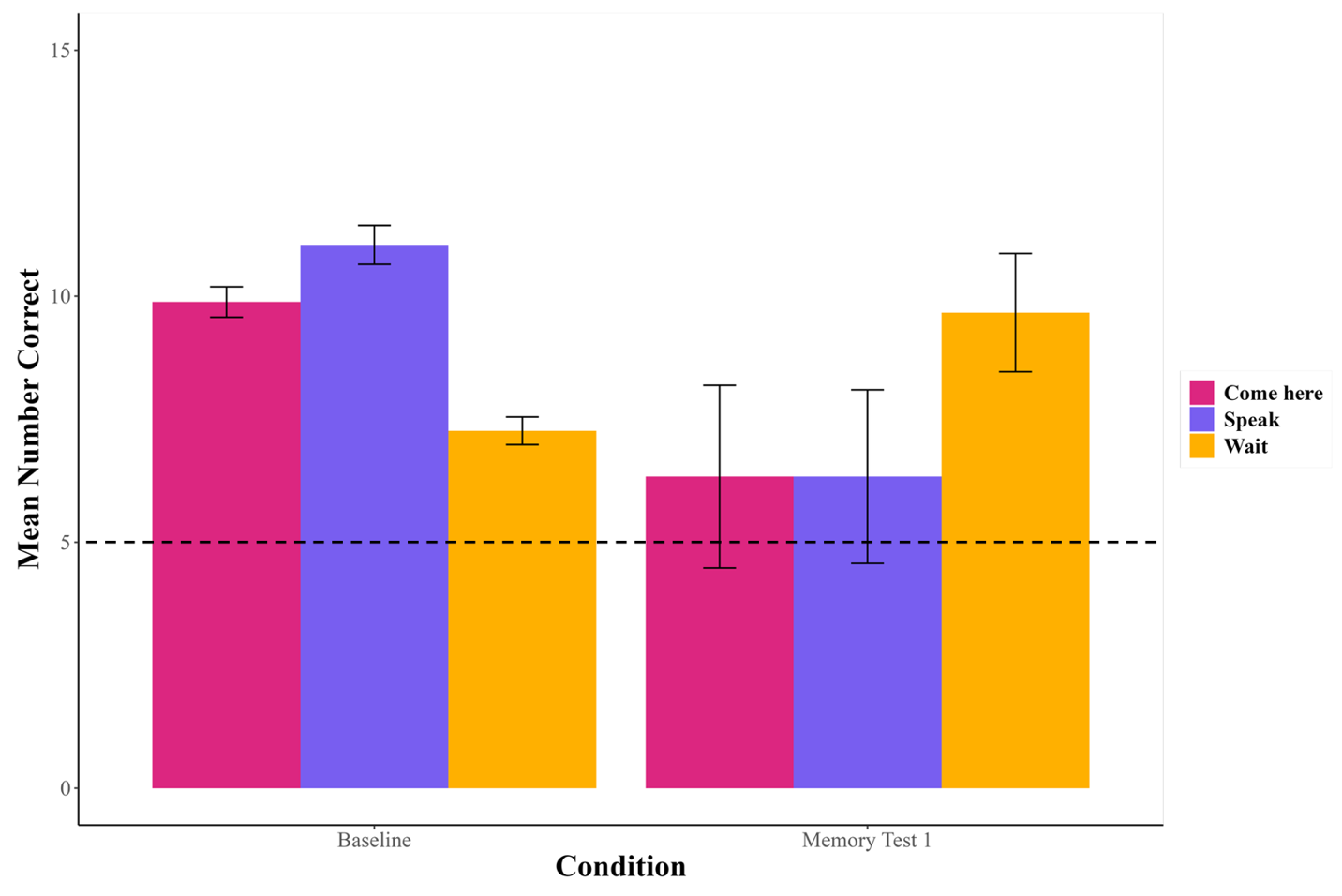


**Supplementary** **Fig. 4** Connelly’s average number of correct responses for each command, for each condition experienced. Note that his Memory Test occurred during his Baseline testing, and was unrehearsed (see *Results*). The black dotted line represents chance at 1/3

*Overall Performance:*

In order to examine the factors affecting Connelly’s accuracy on the commands, after a Box-Cox transformation on the data, an ANOVA assessing the effect of the condition, the command type, the progression of time in number of rounds, and their interactions was carried out (*Supplementary* *Table 6*). There was no significant effect of the interaction of condition and number of rounds. However, there were significant effects of the command type, the condition, the interaction of command type and number of rounds, the interaction of command type and condition, and of the progression of time in number of rounds. A post-hoc Tukey HSD test on the effect of the command type detected significant differences between “Wait!” and “Come here", “Wait!” and “Speak”, and “Come here” and “Speak” (see *Supplementary* *Table 7*). A post-hoc Tukey HSD test on the effect of the condition detected significant differences between the Baseline and the Memory Test (p = 0.012). A post-hoc Tukey HSD test on the effect of the interaction of command type and condition detected some significant differences of interest (differences between, for instance, “Speak” for the Baseline and “Come here” for the Memory Test were not considered to be of relevance, see *Supplementary* *Table 8*). A post-hoc linear mixed-effects model on the effect of the progression of rounds found a significant increase in number of correct choices for increasing rounds (0.048±0.011, t = 4.398, p < 0.001). A post-hoc linear mixed-effects model on the effect of the interaction of command type and progression of rounds, with “Come here” as the reference, detected a significant increase in performance for “Speak” as rounds progressed (0.091±0.020, t = 4.551, p < 0.001), but also a significant decrease in performance for “Wait!” as rounds progressed (-0.090±0.020, t = -4.491, p < 0.001), compared to “Come here”, which had a significant increase in performance as rounds increased (0.048±0.014, t = 3.361, p < 0.001). Therefore, although there is some evidence of learning from Connelly as sessions went on, he appeared to get worse at “Wait!” over time, compared to the other two commands. He also did not reach criterion in the time he was tested. Additionally, he was significantly worse at “Wait!” than the other two commands, similarly to the other rooks, but unlike them, he was also significantly better at “Speak” than “Come here”.


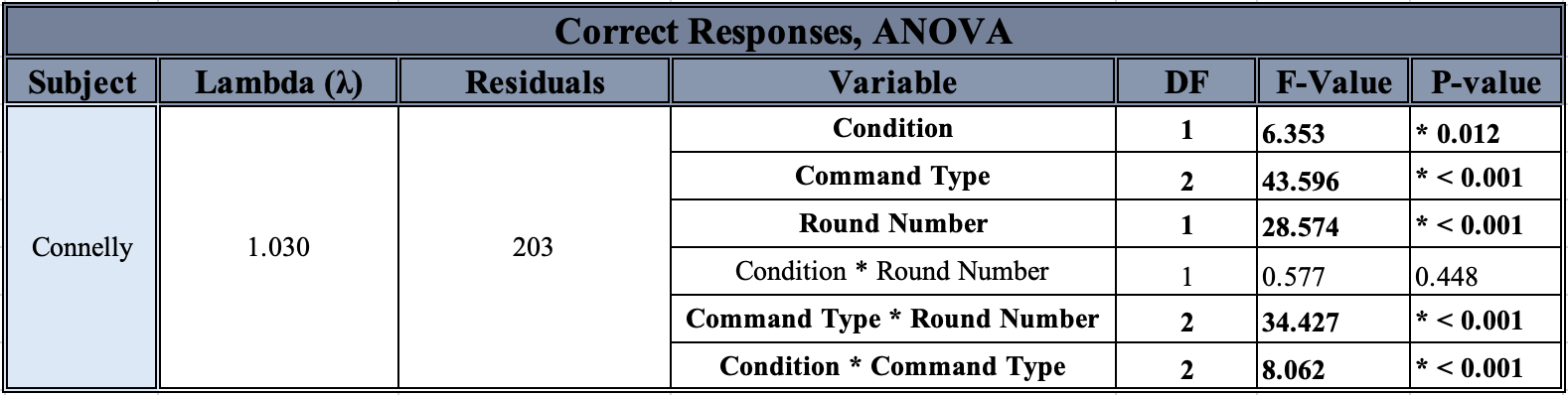


**Supplementary Table 6** ANOVA output for the effect of various variables of interest on Connelly’s performance. Significant results are denoted by an asterisk and bold lettering


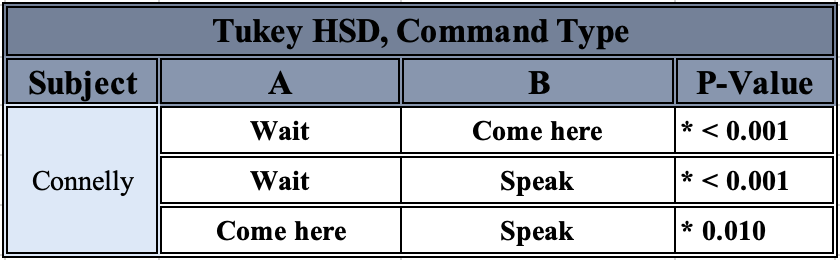


**Supplementary Table 7** Post-hoc Tukey HSD results for the effect of command type on Connelly’s performance. Significant results are denoted by an asterisk and bold lettering


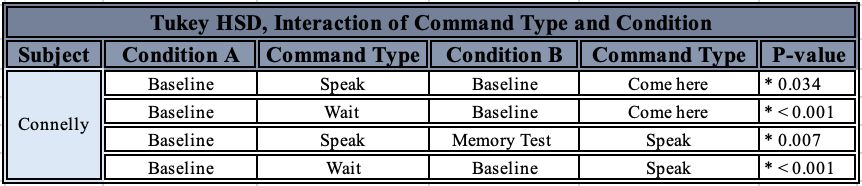


**Supplementary Table 8** Post-hoc Tukey HSD results for the effect of the interaction of condition and command type on Connelly’s performance. Only significant results of interest (those excluding completely mismatched categories) are listed

All Individuals:

*Learning Rates:*

Leo’s learning rate during the Baseline was compared to that of Fry and Connelly. Of note, Leo was more accurate when he began his Baseline (likely due to more extensive training and prior experience with the commands, see *Methods*) than either Fry or Connelly. At the beginning of the Baseline, Leo was 10/15 on “Wait!”, 10/15 on “Speak”, and 11/15 on “Come here”. On the other hand, Fry did not achieve 31/45 correct on all commands in any combination at any point during her Baseline training (her highest achievement was 28/45 correct, on Round 16, with 5 correct at “Wait!”, 11 correct at “Speak”, and 12 correct at “Come here”). Connelly did not achieve 31/45 correct on all commands in any combination until Round 27, with 9 correct at “Wait!”, 12 correct at “Speak”, and 10 correct at “Come here” (his highest achievement was 37/45 correct, on Round 66, with 11 correct at “Wait!”, 14 correct at “Speak”, and 12 correct at “Come here”). Accordingly, and with the small number of trials carried out with Fry in particular, comparisons between individuals are very tentative.

A linear regression was fitted for each individual bird, investigating the effect of the number of rounds conducted on the number of correct responses for all three main commands (see *Supplementary* *Figure 5*). For Leo, a significant learning rate of 0.056±0.027 was identified (t = 2.104, p = 0.038). Connelly also had a significant learning rate (0.043±0.011, t = 3.967, p < 0.001), as did Fry (0.171±0.083, t = 2.070, p = 0.044). Interestingly, all three intercepts were calculated to be significantly different from 0, even during the first round of learning (Leo: 10.643±0.457, t = 23.284, p < 0.001; Fry: 5.836±0.845, t = 6.903, p < 0.001; Connelly: 7.910±0.431, t = 18.348, p < 0.001). This was unexpected, as an intercept of 0 would indicate new learners having no knowledge of commands, as was thought to be the case for Fry and Connelly (who did not yet appear to associate the behaviors with the words spoken when testing began). However, this estimate suggests instead that some learning had already occurred in pre-training.This is also supported by Leo’s intercept being the highest, which corresponds with his greater prior experience).


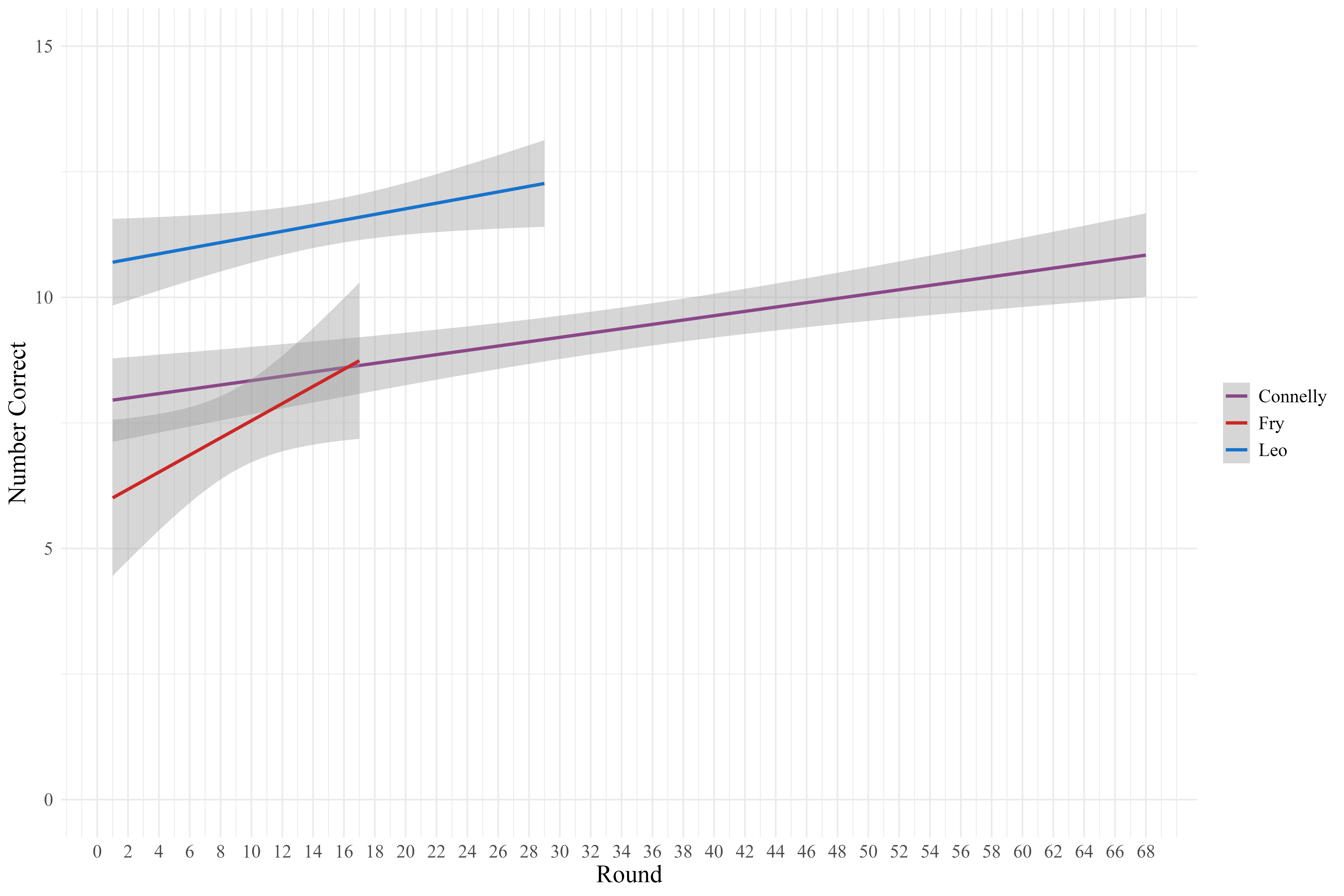


**Supplementary Fig. 5A** Linear regression learning curves for each individual for the Baseline condition, with standard error


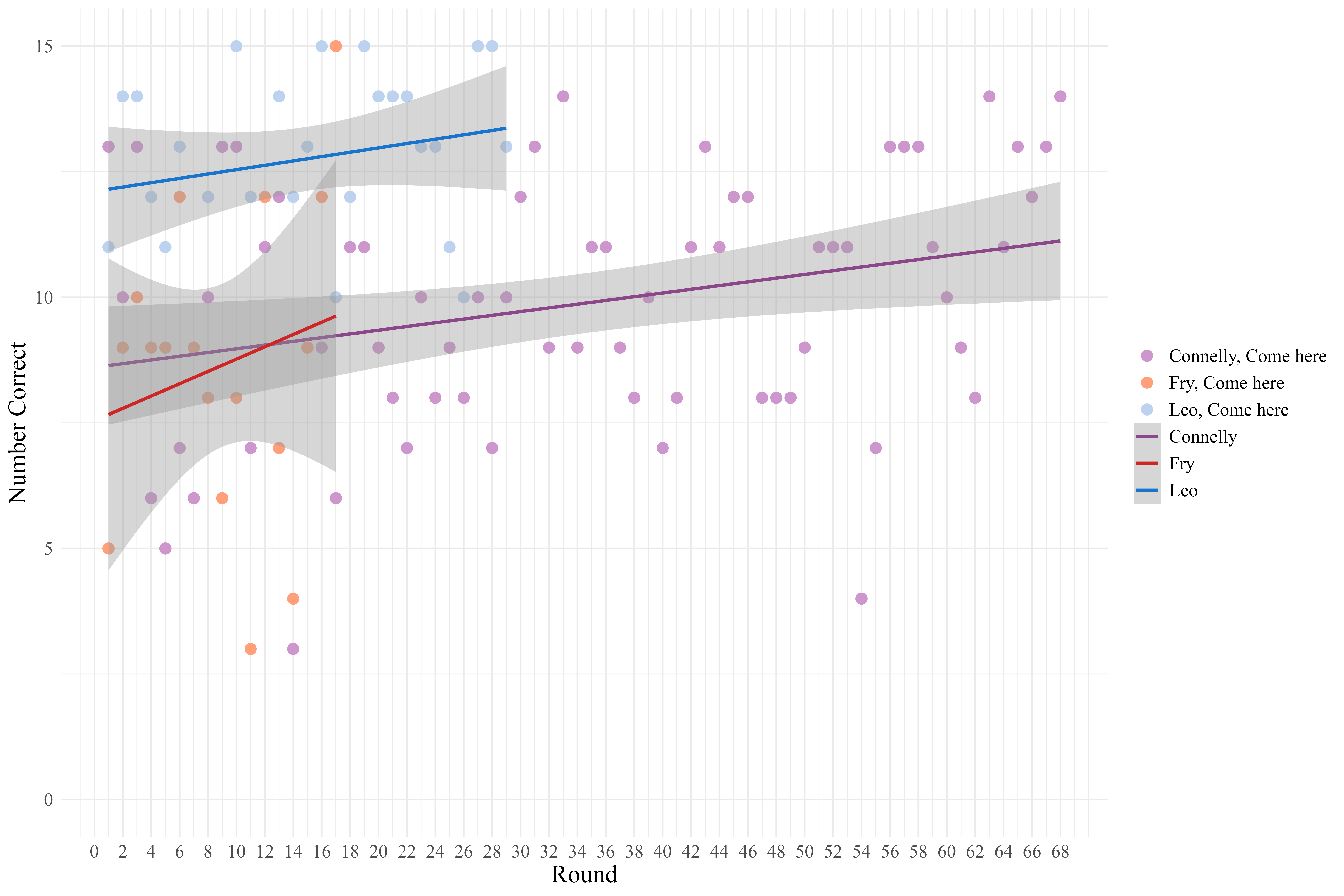


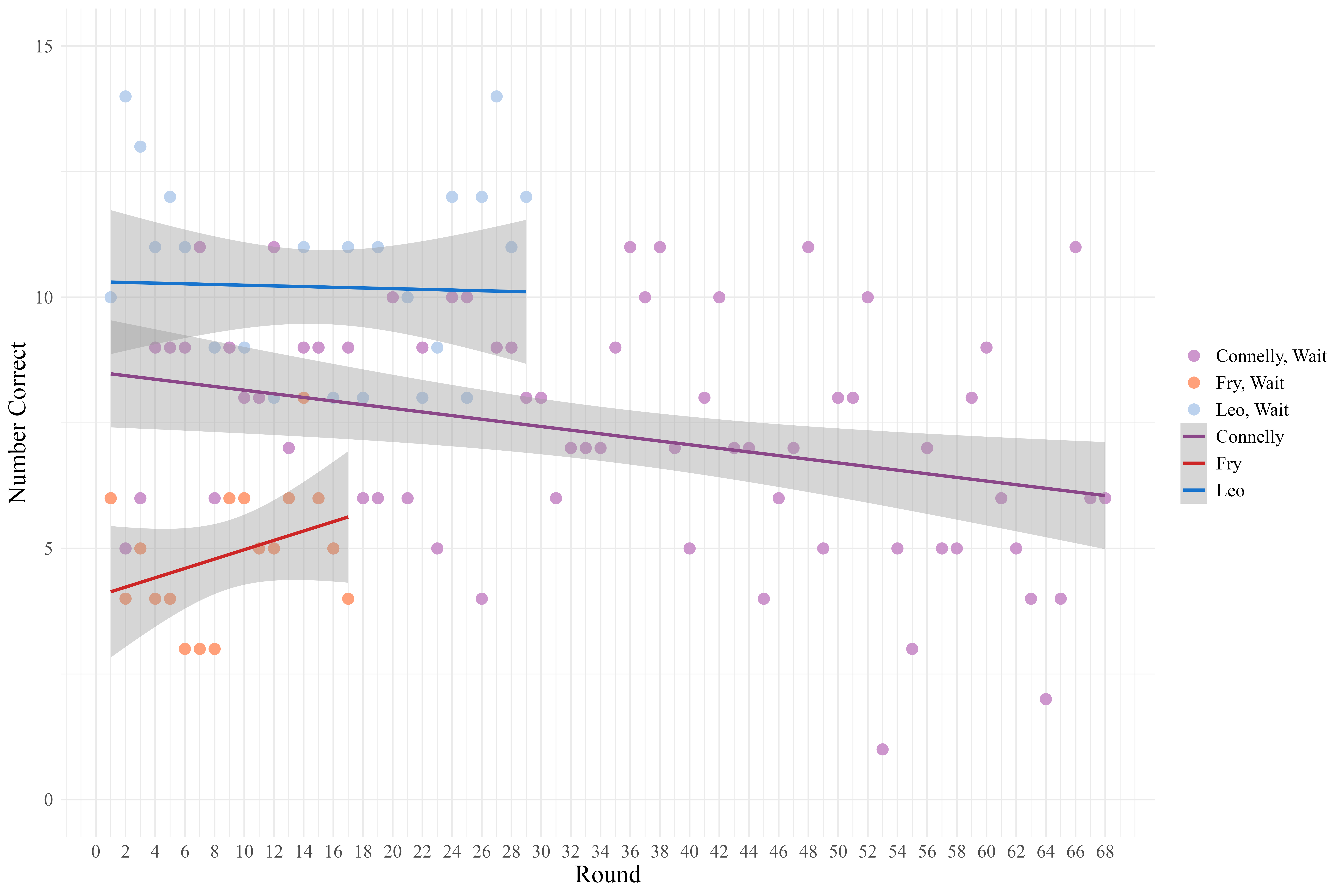


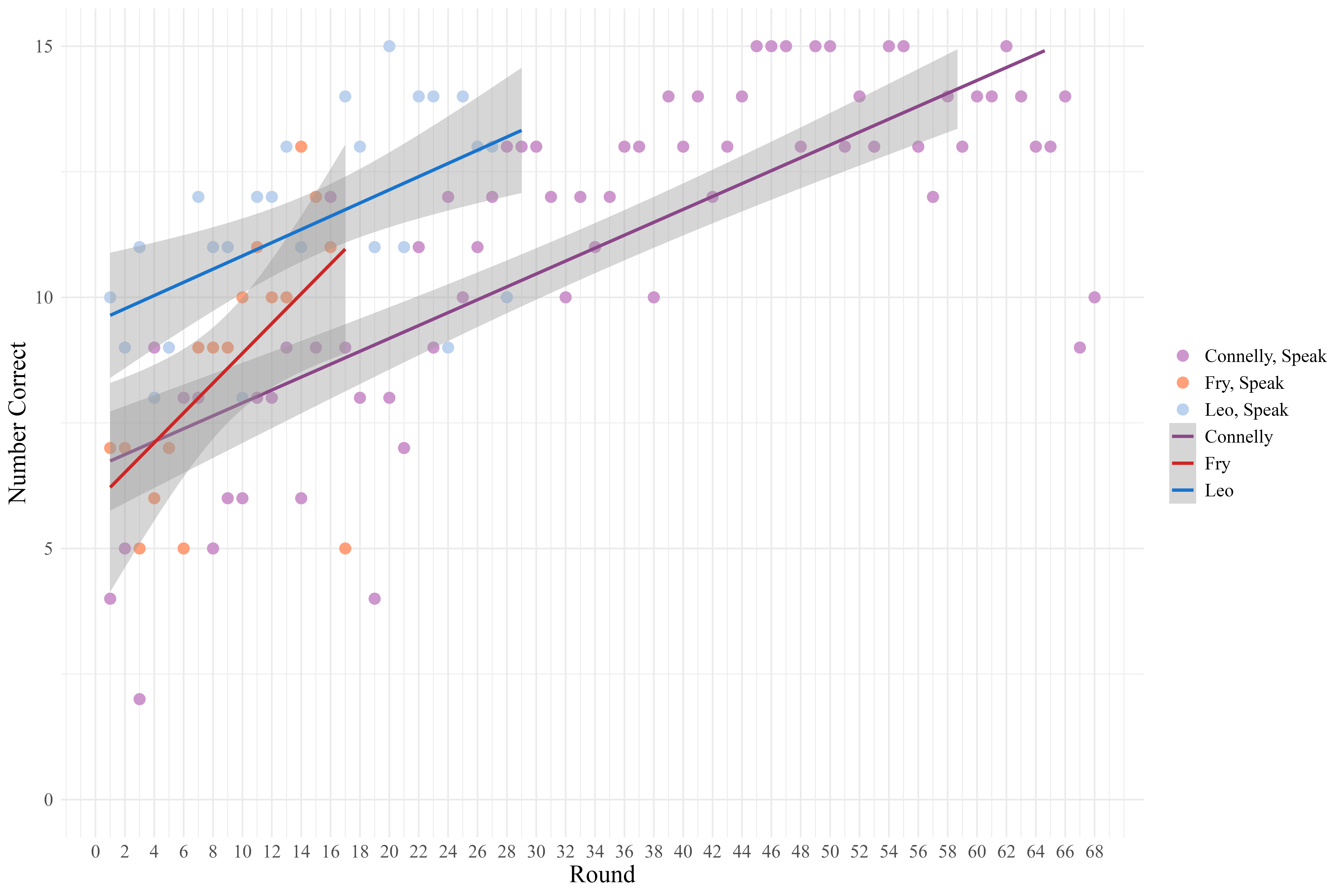


**Supplementary Fig. 5B** Linear regression learning curves for each individual for the Baseline condition, with standard error. Each command is plotted separately: “Come here” (*top*), “Wait” (*middle*) and “Speak” (*bottom*)

*Tentative Discussion, Learning Rates:*

In terms of learning rates, Fry appeared to be learning qualitatively faster than Connelly and Leo, at least during the rounds she participated in; Connelly’s learning rate was nearly identical to Leo’s learning rate, but these comparisons cannot be assumed to be meaningful, since Leo had much more prior experience with the commands and started at a higher level of proficiency than either Fry or Connelly. Fry’s apparent faster rate of learning, too, might be an artifact of reducing performance to a constant linear regression. Her higher learning rate may appear so because faster learning may occur initially and she received less trials compared to Leo and Connelly, whereas when birds are closer to reaching proficiency, performance increases may slow and cause the line to appear flatter overall. Alternatively, individuals’ learning rates may vary, and Fry might have learned faster than the other birds had she continued. However, because both Fry and Connelly never formally reached the required high level of profiency at the commands to move on to formal testing, and because Fry in particular had a much more limited number of sessions given, the learning rates obtained from these birds must be interpreted with caution. Regardless, learning was occurring as demonstrated by the significant effects of the progression of rounds over the Baseline condition for all birds, and generally throughout the experiment for Leo (although his need to return to a very high criterion to pass on to the next condition likely ensured this was the case).
